# Circular RNA SAMD4A controls adipogenesis in obesity through the miR-138-5p/EZH2 axis

**DOI:** 10.7150/thno.42417

**Published:** 2020-03-26

**Authors:** Yanjun Liu, Hongtao Liu, Yi Li, Rui Mao, Huawu Yang, Yuanchuan Zhang, Yu Zhang, Pengsen Guo, Dafang Zhan, Tongtong Zhang

**Affiliations:** 1 Center of Obesity and Metabolic Diseases, The Third People's Hospital of Chengdu, Chengdu, 610031, China.; 2 Affiliated Hospital of Southwest Jiaotong University, Chengdu, 610036, China.; 3 Department of Radiology, The Third People's Hospital of Chengdu, Chengdu, 610031, China.; 4 Medical Research Center, The Third People's Hospital of Chengdu, Chengdu, 610031, China.; 5 The Second Affiliated Hospital of Chengdu, Chongqing Medical University, Chengdu, 610031, China.

**Keywords:** circSAMD4A, obesity, adipogenesis, miR-138-5p, EZH2

## Abstract

A growing body of evidence has suggested that circular RNAs (circRNAs) are crucial for the regulation of gene expression and their dysregulation is implicated in several diseases. However, the function of circRNAs in obesity remains largely unexplored.

**Methods**: Global changes in the circRNA expression patterns were detected in adipose tissues derived from obese and lean individuals. In particular, circSAMD4A was identified as significantly differentially upregulated and was functionally analyzed, both in vitro and in vivo, using various approaches.

**Results**: CircSAMD4A overexpression was correlated with a poor prognosis in obese patients. By contrast, circSAMD4A knockdown inhibited differentiation in isolated preadipocytes. In high-fat diet (HFD) -induced obese mice, circSAMD4A knockdown reversed the associated weight gain, reduced food intake, lower body fat, and increased energy expenditure. These mice also exhibited increased insulin sensitivity and glucose tolerance. Furthermore, in vitro experiments indicated that circSAMD4A affected differentiation by binding to miR-138-5p and regulating EZH2 expression.

**Conclusions**: CircSAMD4A regulated preadipocyte differentiation by acting as a miR-138-5p sponge, and thus increasing EZH2 expression. These results suggested that circSAMD4A can serve as a potential target for obesity treatments and/or as a potential prognostic marker for obese patients following bariatric surgery.

## Introduction

Obesity, one of the greatest worldwide health burdens, is a major cause of many chronic illnesses, including cardiovascular disease, type 2 diabetes, and cancer 1,2. Due to recent nutritional habits, the incidences of obesity are on the rise 3.Thus, to effectively combat obesity, it is necessary to identify its associated molecular mechanisms and critical signaling pathways.

Several studies have highlighted the regulatory mechanisms by which non-coding RNAs (ncRNAs), including microRNAs (miRNAs) and long non-coding RNAs (lncRNAs), participate in the development of many diseases, such as obesity 4,5. While, miRNAs and lncRNAs have been examined in association with obesity 6,7, the third class of ncRNAs, known as circular RNAs (circRNAs), has attracted research interest only recently 8. CircRNAs have been implicated in gene regulation in a wide variety of organisms 9. However, the mechanisms by which these ncRNAs function during disease progression have not been clearly elucidated. It has been suggested that circRNAs may regulate gene expression via different targets in different types of diseases or even at different disease stages 8. Furthermore, emerging evidence has suggested that some circRNAs act as miRNA sponges by modulating gene transcription and interacting with RNA binding proteins (RBPs) in various diseases, including cancer 10,11, cardiovascular disorder 12, and immunosenescence 13. In the adipose tissue, several studies have indicated that circRNAs function as important regulators in adipogenesis 14, adipose inflammation 15, and white adipose browning 16. However, the regulatory mechanisms of circRNAs in adipogenesis are not clearly understood.

In this study, circRNA expression profiles were constructed for adipose tissue samples from obese and lean individuals using circRNA microarrays. In obese individuals, circSAMD4A (hsa_circ_0004846) was significantly upregulated and was related to the prognosis. Functional assays indicated that circSAMD4A knockdown inhibited preadipocyte differentiation. Furthermore, circSAMD4A expression was positively correlated with EZH2 expression in obese individuals. The results presented herein show that circSAMD4A binds to miR-138-5p and acts as a miRNA sponge to subsequently regulate EZH2 expression. Overall, these findings suggest that circSAMD4A also acts as an adipogenesis promotion factor, and can serve as a prognostic biomarker for obesity.

## Materials and Methods

### Clinical samples

Adipose tissue samples were prospectively collected from 40 patients undergoing laparoscopic hernia repair [in lean (Ln) volunteers] and 60 patients undergoing bariatric surgery [in obese (Ob) subjects] at the Third People's Hospital of Chengdu, China between December 2016 and November 2017 (Table S1). According to the China National Nutrition and Health Survey (CNNHS) data, a BMI of ≥28 kg/m^2^ in Chinese adults suggests obesity 17. Microarray analyses (targeting circRNAs) were performed on the tissue samples of the human. This study was approved by the Institutional Ethics Review Board of the Third People's Hospital of Chengdu (record #: 2018S75; Chengdu, Sichuan, China), and was conducted in accordance with the Chinese ethical guidelines for human genome/gene research.

### CircRNA microarray and computational analyses

Total RNAs were digested with Rnase R (Epicenter Technologies, Madison, WI, USA) to remove linear RNAs and enrich circular RNAs according to the manufacture's protocols. The enriched circular RNAs were then amplified and transcribed into fluorescent cRNA utilizing a random priming method. The labeled cRNAs were hybridized onto the Human circRNA Array (4 × 180K, Shanghai Biotechnology Corporation, Shanghai, China) containing 4 identical arrays with probes slightly under 180K. The arrays were then washed and scanned, using an Agilent scanner and the images were analyzed using fold-change filtering and volcano plots, with a fold-change ≥1.5 and a P-value <0.05 considered significantly differentially expressed. To determine if the obtained circRNA profiles were distinguishable between the two groups, hierarchical clustering was performed to test whether the two types of samples were distinguishable based on circRNA expression patterns. The obtained circRNA microarray datasets were deposited with the NCBI Gene Expression Omnibus (GEO) repository under accession number GSE131819.

### Quantitative real-time PCR (qRT-PCR)

Total RNAs were reverse-transcribed into cRNA with random primers using Transcriptor First Strand cDNA Synthesis Kit (Roche, Penzberg, Germany) according to the manufacturer's instructions. CircSAMD4A expression was quantified via qRT-PCR using the FastStart Essential DNA Green Master (Roche, Penzberg, Germany) on a Roche LightCycler 480 (Roche, Penzberg, Germany). Relative levels were determined using the 2-ΔΔCt method, with circRNA levels normalized to GAPDH. Divergent primers, rather than the more commonly used convergent primers, were designed to target the circRNAs broadly (Table S2). Primer specificity was verified using BLAST, with a single-peak in the melt-curve indicating the generation of a specific product. Experimental triplicates were performed for each sample. Relative expression was determined using inter-experiment normalization to GAPDH. CircRNAs of the obese sample with the lowest expression level was defined as 1.

### Preadipocyte isolation and differentiation

Preadipocyte were isolated from visceral adipose tissues (VAT) and cultured as previously described 18 Briefly, adipose tissue samples were digested with collagenase to obtain stromal cells, which were then separated from the mature adipocytes by centrifugation and incubated in erythrocyte lysing buffer for 10 min at room temperature to eliminate red blood cells. The cellular debris was removed by filtering the cell suspension through a 0.07 mM nylon filter via centrifugation. Pelleted preadipocytes were plated in a basal medium consisting of DMEM/F-12 (Gibco, Carlsbad, CA, USA) supplemented with 10% fetal calf serum (FCS) and incubated for 16-18 h. After the incubation, the attached cells were washed thoroughly with warm phosphate-buffered saline (PBS), removed from plates with trypsin, re-suspended, and counted. At 2 days post-confluence, cells were incubated with induction medium supplemented with 0.005 mg/ml insulin (MedChem Express), 0.001 mM dexamethasone, and 0.5 mM methylisobutylxanthine for 48 h to promote differentiation. Next, the induction medium was replaced with DMEM containing 0.005 mg/ml insulin and then cells were incubated for an additional 48 h. Cells were maintained in DMEM containing 10% FBS until most of the cells had differentiated into mature adipocytes with abundant lipid droplets.

### Prediction miRNA binding potential

Potential miRNA binding sites on circSAMD4A were identified using the miRcode (http://www.mircode.org/mircode) predictive algorithm from the Shanghai Biotechnology Corporation (Shanghai, China) 19. The identified miRNAs were then ranked based on their predicted binding scores.

### CircRNA immunoprecipitation (circRIP) assay

Biotin-labeled circSAMD4A probe was synthesized by GenePharma (Shanghai, China), and a circRIP assay was performed as previously described 20. Briefly, preadipocytes were washed with ice-cold PBS, fixed using formaldehyde, lysed in the co-IP buffer and sonicated. After centrifugation, the supernatant was combined with streptavidin Dynabeads M-280 (Invitrogen, Waltham, MA, USA) and incubated at 30°C for 12 h. Next, the probes-dynabeads-circRNAs mixture was washed and incubated with lysis buffer and proteinase K. Finally, the mixture was combined with Trizol reagent (Invitrogen, Carlsbad, CA, USA) for RNA extraction and detection.

### CircRNA knockdown and miRNA overexpression

Small interfering RNAs (siRNAs) designed to target circRNAs were obtained from GenePharma (Shanghai, China), and were transfected into cells using Lipofectamine 2000 (Invitrogen, Carlsbad, CA, USA). The samples were incubated for 48 h, and circSAMD4A expression was measured using qRT-PCR as described above. The most efficient sequence for hsa_circ_0004846 siRNA was 5'-AGCAC AAGUA CAAGA AUCAU UdTdT-3'. All the miRNA mimics and inhibitors were synthesized by GeneCopoeia (Rockville, MD, USA).

### Immunohistochemistry

VAT samples were fixed in 10% formaldehyde, embedded in paraffin wax, and sectioned (4 μm thick). The samples were then deparaffinized in xylene and rehydrated prior to antigen retrieval. Endogenous peroxidase activity was blocked using 0.3% H_2_O_2_ and then the sections were incubated with rabbit anti-EZH2 (1:1000; Cell Signaling Technology, Danvers, MA, USA) at 4ºC overnight. The sections were washed and incubated with biotinylated secondary antibody and stained with 3,3' diaminobenzidine (DAB). The percentage of immunoreactive cells was scored as follows: 10-25% as a 1, 26-50% scored as a 2, 51-75% scored as a 3, and 76-100% scored as a 4. Staining intensity was scored as follows: no staining scored 0, weak staining scored as 1, moderate staining scored as 2, and strong staining scored as 3. The final staining score was the product of the immunoreactive score and the staining intensity score. Based on the final staining score, samples were divided into a low expression group (final score ≤ 6) and a high expression group (final score > 7).

### Plasmid constructs

CircSAMD4A was amplified from human genomic DNA and cloned into a pCD-ciR vector (Geenseed Biotech Co, Guangzhou, China) which containing a front circular frame. Site directed mutagenesis was performed using a Fast Site-Directed Mutagenesis Kit (Takara Bio Inc., Dalian, China) to target miRNA binding sites on circSAMD4A. All of the constructs were confirmed by sequencing.

### Western blotting

Proteins were extracted from a cell line as well as adipose tissues using RIPA lysis buffer and the bicinchoninic acid protein assay kit (Sigma) was used to determine protein concentrations. The extracts were resolved via 12% SDS-PAGE, and transferred to PVDF membranes. After blocking for 1 h, the membranes were incubated with the primary anti-EZH2, C/EBP-α, PPAR-γ or AGO2 antibodies (1:000; Cell Signaling Technology, Danvers, MA, USA) at 4ºC overnight, followed by a 2 h incubation with HRP-conjugated secondary antibody at room temperature. The immunoreactive bands were visualized using ECL and normalized to GAPDH (the internal control).

### Fluorescent *in situ* hybridization

RNA fluorescent *in situ* hybridization (RNA-FISH) was performed following the instructions of the probe manufacturer's instructions (RiboBio, Guangzhou, China; Table S3). Preadipocytes were sequentially treated with 70%, 85%, and absolute ethanol, and dried at 2°C. Cells were then permeabilized with 0.1%TritonX-100 and incubated with 0.02 mg/ml of circSAMD4A probe overnight at 37°C. The nuclei were stained with DAPI and intracellular localization of circSAMD4A was observed by using a TCS SP8 X laser confocal microscope (Leica).

### *In situ* hybridization (ISH)

CircSAMD4A expression was examined in adipose tissues using ISH with a specific digoxin-labeled circSAMD4A probe (Table S3) obtained from Superchip Biotech (Shanghai, China). Staining and expression levels were evaluated, and scores were assigned. Cell staining scores were assigned as follows: 10-25% stained, score = 1, 26-50% stained, score = 2, 51-75% stained, score = 3, and 76-100% stained, score = 4. Staining intensity was scored as follows: no staining scored as 0, weak staining scored as 1, moderate staining scored as 2, and strong staining scored as 3. The final staining score reflects the product of the staining score and the staining intensity score, with samples divided into a low expression (final score ≤ 6) and a high expression group (final score > 7).

### Luciferase reporter assay

A wild-type circSAMD4A sequence was cloned into a pmiR-RB-Report vector (Ribobio Co., Guangzhou, China), while simultaneously generating mutants using site-directed mutagenesis as described above. The mutations were confirmed via sequencing with vectors containing a mutation sequence used as a negative control. Preadipocytes were seeded in 96-well plates at a density of 4 × 10^3^ cells per well 24 h before transfection. The cells were then transfected with either the wild-type or mutated reporter vectors with lysates obtained at 24 h post-transfection. The Dual-Glo Luciferase Reporter System (Promega, Madison, WI) was used to perform the dual-luciferase assay according to the manufacturer's protocols.

### Oil Red O staining

To visualize intracellular lipid deposits, Oil Red O staining was employed. At different time points after the medium change wild type, preadipocytes were stained with 30% Oil Red O in isopropanol for 60 min. Light microscopy was used to identify lipid deposits and confirm differentiation.

#### Animals

Male C57BL/6J mice (9-weeks-old) were kept in a pathogen-free facility and maintained under a 12 h light-dark cycle at 22°C. Mice were fed *ad libitum* with a high-fat diet (HFD; TD88137 Harlan Teklad). To obtain tissue samples, mice were fasted for 16 h and subsequently anesthetized with inhalational anesthetic isoflurane and decapitated. Tissues of interest were excised and kept at -80°C or in formalin until analysis. Animal care and experimental procedures were approved by the Ethics Committee in Animal Experimentation of West China Hospital, Sichuan University, Chengdu, China (record #: 2019014A).

### Adeno-associated virus preparation and injection

AAV9 was designed to target mmu_circ_0000529 by SunBio (Shanghai, China) and scrambled non-targeting shRNA was used as a control. The mmu_circ_0000529 siRNA sequence was as follows: 5'-GGCGC AAGCA CGAGA AUCAU UdTdT-3'. Mice were anesthetized with isofluorane (1-4%), and placed in a prone position. The virus was diluted in sterile PBS (1 × 10^12^ vg/ml) and multi-point injections were administered intraperitoneally or subcutaneously using an insulin syringe.

### Glucose and insulin tolerance tests

Glucose and insulin tolerance tests (GTT, ITT) were performed by intraperitoneally injecting glucose (2 g/kg, 20% wt/vol d-glucose [Sigma] in 0.9% wt/vol saline) or insulin (0.75 unit/kg in 0.9% wt/vol saline) to mice that had been fasted for 6 h. Blood glucose levels were measured at 0, 15, 30, 60, and 120 minutes using an Infinity glucose meter (US Diagnostics).

### Body Mass and Composition Measurements

Mice were weighed on an electronic scale. Body composition was determined by using time domain-nuclear magnetic resonance (TD-NMR) on a Minispec Analyst AD lean/fat analyzer (Bruker Optics, Silberstreifen Germany).

### Energy expenditure

To measure food intake and metabolic rates in the mice, an Oxymax open-circuit indirect calorimetry (16-chamber; Columbus Instruments, Columbus, OH, USA) was used. Briefly, mice were individually housed in calorimeter chambers through which air with a known O_2_ concentration and air passed at a constant flow rate. The system automatically withdrew gas samples and calculated the volumes of O_2_ consumed and the volume of CO_2_ for each mouse. Heat expenditure was measured throughout during both light and dark cycles, and food consumption measurements were based on breaking a horizontal photobeam.

### Statistical analyses

All the statistical analyses were performed with SPSS v20.0 (SPSS, Inc., Chicago, IL). A Student's *t-*test was used to compare the two groups. The *chi*-squared test was used to identify significant correlations between circSAMD4A expression and clinical-pathological features associated with the obese patient samples. The ROC analysis was used for testing if the survival prediction was sensitive and specific based on circRNA risk score. The calculation of area under the curve (AUC) values was carried out according to ROC curves. Multivariate logistic regression was used to estimate odds ratios for the potential predictors of 1-year non-remission. *P* < 0.05 was considered significant.

### Ethics statement

The use of the patient samples in this study was approved by the Institutional Ethics Review Board of Chengdu Third People's Hospital (Chengdu, Sichuan, China), and was conducted according to the Ethical Guidelines for Human Genome/Gene Research issued by the Chinese Government. The ethical record number was 2018S75. Animal care and experimental procedures were approved by the Ethics Committee in Animal Experimentation of West China Hospital, Sichuan University. The ethical record number was 2019014A.

## Results

### Differential circRNA expression in adipose tissues from obese and lean individuals

**Figure 1:**
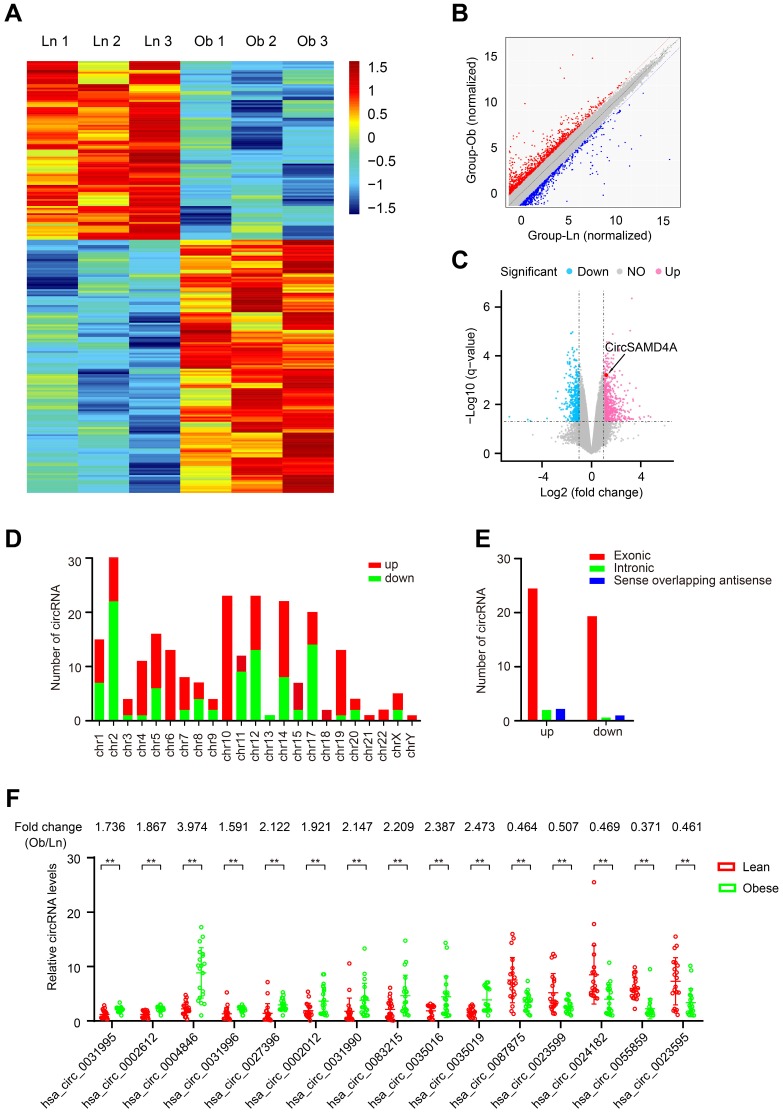
** Identification of differentially expressed circRNAs in obese patients. (A)** Clustered heatmap of the differentially expressed circRNAs in VAT samples from 3 obese and 3 lean patients. Upregulated circRNAs are shown in red and downregulated circRNAs are shown in green. **(B)** Scatter plot showing differences in circRNA expression between the adipose tissues of obese patients and those of lean patients. The red dotted line indicated upregulation by 1.5-fold and the green line indicated downregulation by 1.5-fold. **(C)** Volcano plots comparing circRNA expression between obese and lean patients. The horizontal line corresponds to a P value of 0.05, and the vertical lines indicate upregulation and downregulation by 1.5-fold. The cyan and purple points indicate the differentially expressed circRNAs with a greater than 1.5-fold change between the two compared groups. **(D)** Numbers of identified circRNAs from different chromosomes. **(E)** Genomic origins of the differentially expressed circRNAs in the obese patients. **(F)** Differential expression of 15 circRNAs was validated in 20 adipose tissues from obese patients and 20 adipose tissues from lean patients using qRT-PCR. Data are presented as means ± SD; significant difference was identified with Student's t test. *P < 0.05; **P < 0.01; ns (not significant).

CircRNA expression profiles were constructed for both obese and lean VATs (n = 6) using circRNA microarrays. The circRNA profile dataset was visualized using a heatmap (Figure 1A). After normalization, log2 ratio distributions were examined among the six samples, which were found to be very similar. The degree of variation between the two groups was evaluated using a scatter plot (Figure 1B), and differentially expressed circRNAs that were significantly different between the two groups were identified using volcano plot filtering (Figure 1C). In the samples from obese VATs, 244 significantly differentially expressed circRNAs were identified with significant differential expression relative to the lean VAT samples (*P* <0.05 and *q* <0.05) (Table S2) with 143 upregulated and 101 downregulated. The chromosomal distribution of the differentially expressed circRNAs was examined (Figure 1D) and the genomic origins were identified (Figure 1E).

### Characterization of circSAMD4A in preadipocytes

**Figure 2:**
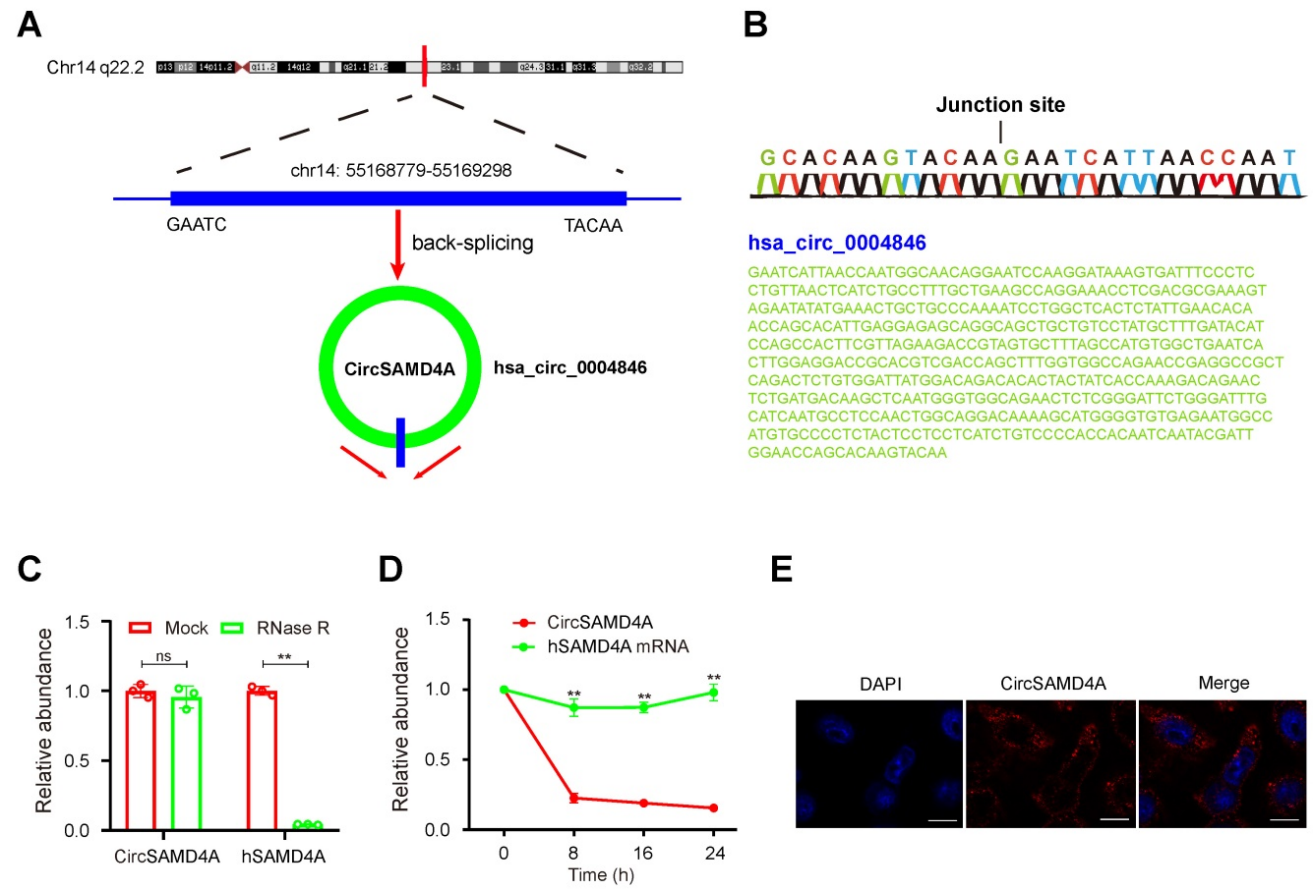
** Characterization of circSAMD4A in preadipocytes. (A)** Genomic location of the hSAMD4A gene and of circSAMD4A. **(B)** Sanger sequencing showing the “head-to-tail” splicing of circSAMD4A in preadipocytes.** (C)** qRT-PCR of quantification of circSAMD4A and hSAMD4A mRNA expression in preadipocytes after treatment with RNase R. **(D)** qRT-PCR quantification of circSAMD4A and hSAMD4A mRNA expression in preadipocytes after treatment with Actinomycin D. **(E)** RNA FISH for circSAMD4A. Nuclei were stained with DAPI. Scale bar = 20µm. Data are presented as means ± SD; significant difference was identified with Student's t test. *P < 0.05; **P < 0.01; ns (not significant).

From the differential expression microarray data, the top 10 upregulated as well as the top 5 downregulated circRNAs with most significant P-values were selected for further investigation (Table S2 and Figure 1C). These circRNAs from 20 obese and 20 lean VAT samples were analyzed by qRT-PCR (Figure 1F). The analysis revealed hsa_circ_0004846 as the most significantly expressed circRNA. Based on the human reference genome (GRCh37/hg19), we hypothesized that hsa_circ_0004846 (chr14: 55168779-55169298) is derived from hSAMD4A, which is located on chromosome 14q22 (Figure 2A). Thus, hsa_circ_0004846 was named “circSAMD4A.” The sequence of circSAMD4A was confirmed with Sanger sequencing (Figure 2B); it was found to be RNase R resistant, thus suggesting a circular configuration (Figure 2C and Figure S1A). To determine the half-life of circSAMD4A, its expression level, together with the hSAMD4A level was examined following actinomycin D transcriptional inhibition. The results indicated circSAMD4A to be more stable than hSAMD4A (Figure 2D). Furthermore, examination with RNA-FISH showed that circSAMD4A was predominantly located in the cytoplasm (Figure 2E).

### Upregulation of CircSAMD4A correlates with an increased body mass index (BMI) and non-remission after bariatric surgery

**Figure 3:**
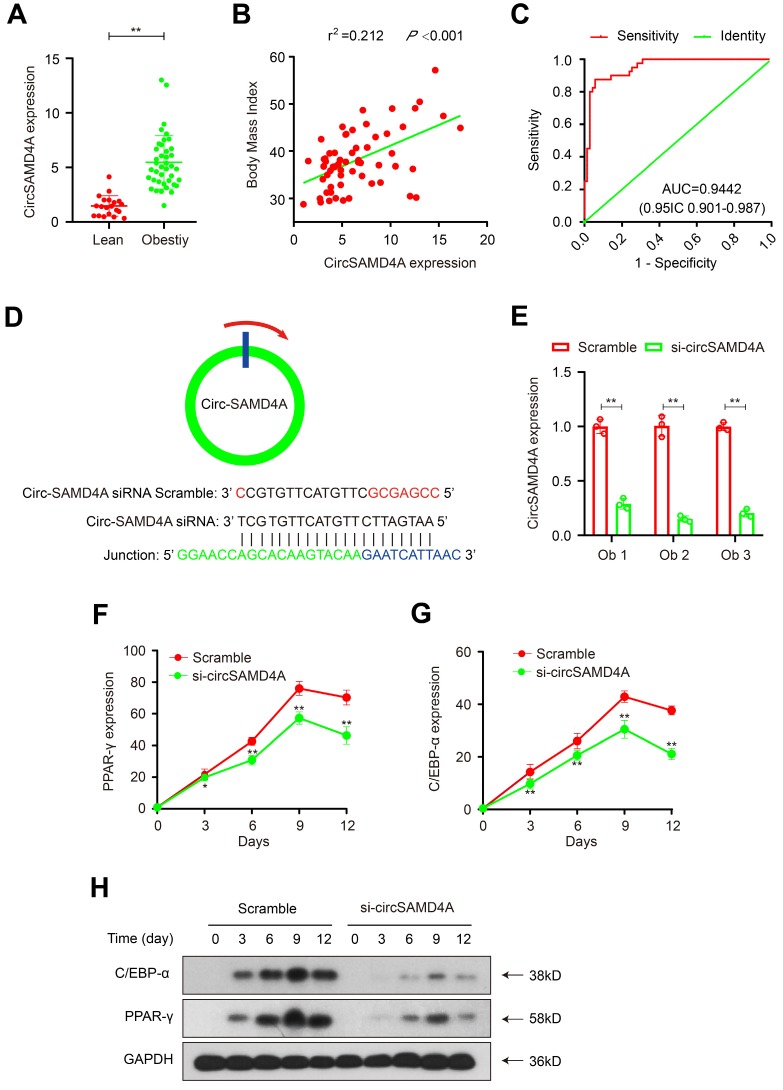
** CircSAMD4A as an independent risk factor that can predict non-remission in the obese patients. (A)** CircSAMD4A expression in adipose tissues from 40 obese patients and 20 lean patients. **(B)** Pearson correlation between circSAMD4A expression and BMI in adipose tissues of 60 obese patients. **(C)** ROC curve for circSAMD4A indicating its diagnostic value in obese patients. **(D)** Schematic representation the siRNA sites specific to the back-splice junction of circSAMD4A. **(E)** Expression of circSAMD4A following siRNA treatment using qRT-PCR. (**F-G**) C/EBP-α and PPAR-γ expression was quantified using qRT-PCR after circSAMD4A knockdown during preadipocytes differentiation. (**H**) C/EBP-α and PPAR-γ expression was quantified using Western blot assay after circSAMD4A knockdown during preadipocyte differentiation. Data are presented as means ± SD; significant difference was identified with Student's t test. *P < 0.05; **P < 0.01; ns (not significant).

To further evaluate the role of circSAMD4A in obesity, a potential correlation with prognosis was examined in another cohort comprising 40 obese and 20 lean patient samples. CircSAMD4A expression levels were quantified using qRT-PCR and found to be significantly upregulated in the obese patients relative to the lean controls (Figure 3A). Next, a potential correlation between circSAMD4A expression and clinicopathological status was examined using a Pearson correlation test, which showed that a positively correlated between circSAMD4A expression and BMI in the 60 obese patients (*P* < 0.01 and r^2^ = 0.212; Figure 3B and Table S4). More importantly, a multivariate logistic regression analysis revealed that increased circSAMD4A expression was significantly associated with non-remission within one year of bariatric surgery (Table S5 and Supplementary Methods). Furthermore, ROC analysis performed with a circSAMD4A cut-off ≥ 2.83 showed a high diagnostic performance, as reflected by the Youden index (sensitivity 93.3% and specificity 87.5%; Figure 3C). Together, these findings suggested that circSAMD4A upregulation in adipose tissues was correlated with a poor prognosis in obese patients, and that monitoring overexpression of circSAMD4A expression might accurately predict obesity outcomes.

### Knockdown of circSAMD4A inhibits preadipocyte differentiation

We isolated preadipocyte from the tissue samples of obese patient and performed circSAMD4A knockdown using siRNAs targeting the back-splice sequence (Figure 3D). Successful knockdown was confirmed using qRT-PCR that showed hSAMD4A expression to be unaffected (Figure 3E and S1B). Furthermore, in circSMAD4A knockdown preadipocytes, Oil Red O staining indicated inhibition of differentiation (Figure S2). Additionally, preadipocyte differentiation biomarkers, C/EBP-α and PPAR-γ 21,22, were decreased in circSAMD4A knockdown cells, as quantified by qRT-PCR and Western blot analysis (Figure 3F-H).

### CircSAMD4A functions as a sponge for miR-138-5p

**Figure 4:**
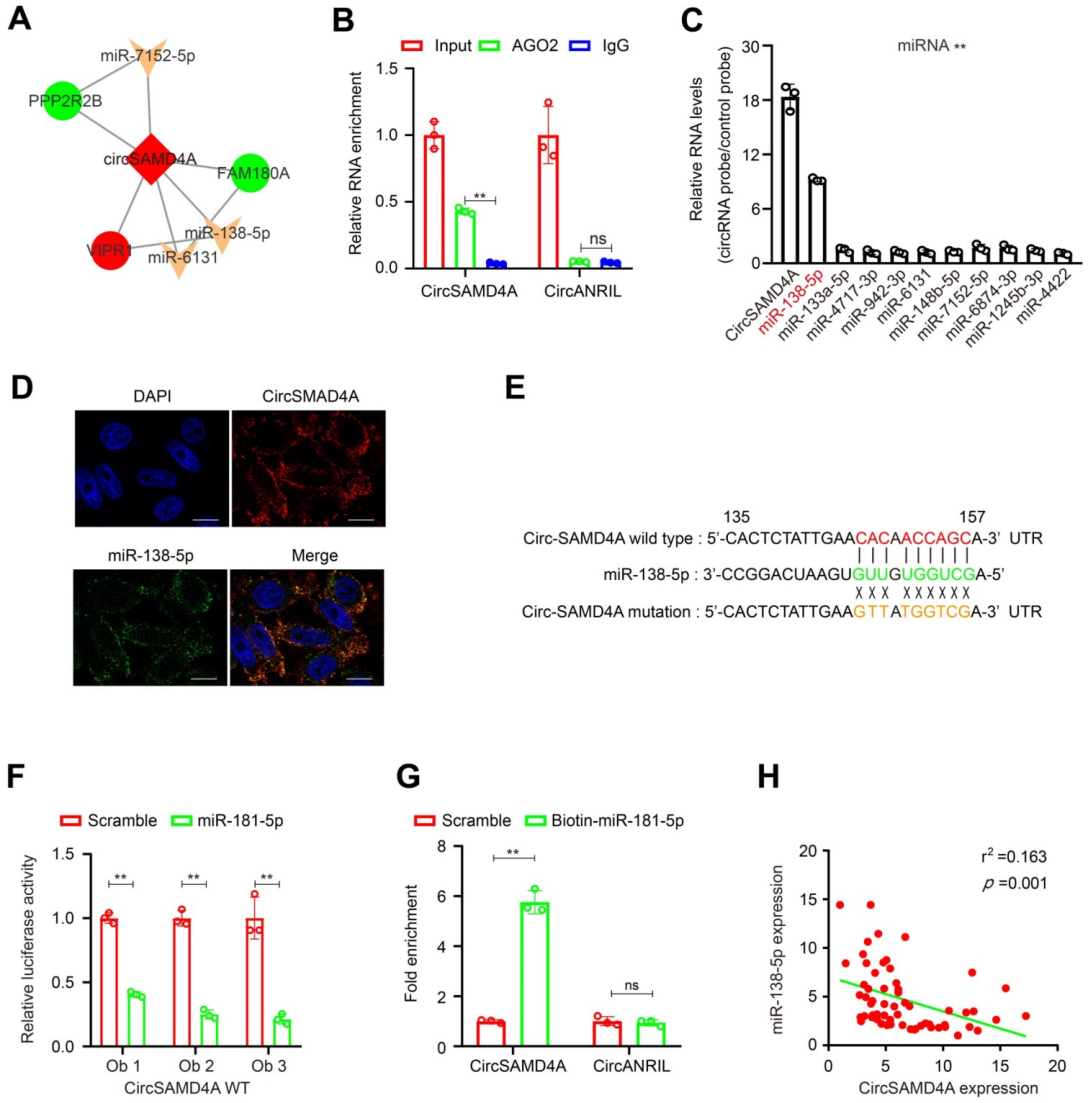
** CircSAMD4A acts as a miRNA sponge for miR-138-5p. (A)** CircSAMD4A-miRNA-mRNA network and pathway analysis. **(B)** RIP experiments were performed using an antibody against AGO2 on extracts from preadipocytes. **(C)** CircRIP was performed using a circSAMD4A-specific probe and control probe in preadipocytes from obese patients. The enrichment of circSAMD4A and microRNAs was detected by qRT-PCR and normalized to the control probe. **(D)** Co-localization between circSAMD4A and miR-138-5p was observed by RNA *in situ* hybridization in preadipocytes. Nuclei were stained with DAPI. Scale bar = 20µm. **(E)** Schematic showing the predicted miR-138-5p sites in circSAMD4A. **(F)** Luciferase assays in preadipocytes co-transfected with a scrambled control, miR-138-5p mimic, and a luciferase reporter plasmid containing either wild-type circSAMD4A (circSAMD4A-WT). **(G)** qRT-PCR showing the level of circSAMD4A in the streptavidin-captured fractions from the preadipocytes lysates after transfection with biotinylated miR-138-5p or control RNA. CircANRIL (a circular RNA reported not to bind to AGO2) was used as a negative control. **(H)** Pearson correlation between circSAMD4A expression and miR-138-5p expression in adipose tissues of 60 obese patients using qRT-PCR. Data are presented as means ± SD; significant difference was identified with Student's t test. *P < 0.05; **P < 0.01.

Previous studies have shown that circRNAs act as miRNA sponges 23, thus the ability of circSAMD4A to bind miRNAs was explored. To identify miRNAs that might potentially bind to circSAMD4A, miRcode was employed identifying 10 potential miRNAs, which were used to construct a circSAMD4A-miRNA-mRNA network (Figure 4A and Table S6). To further explore circSAMD4A associations, a circRIP assay with antibodies against argonaute 2 (AGO2) was performed because of AGO2's role in miRNA-induced RNA silencing in the adipose tissue 20,24. The results showed that the AGO2 antibody significantly enriched circSAMD4A (Figure 4B), but not cicrANRIL a circular RNA with no binding ability to bind to AGO2 20,25, thus suggesting that circSAMD4A acts as a binding platform for AGO2 and miRNAs. Additionally, circSAMD4A associated miRNAs were purified using circRIP with specific probes targeting circSAMD4A. The results showed that circSAMD4A and miR-138b-5p were both enriched in the preadipocytes (Figure 4C). This association was further confirmed using RNA-FISH that illustrated the co-localization of circSAMD4A and miR-138-5p in preadipocytes (Figure 4D).

To examine the predicted circSAMD4A binding sites (Figure 4E and Table S6), a dual-luciferase assay was performed as previously described 26 showing high binding affinity between circSAMD4A and miR-138-3p. Furthermore, miR-138-5p reduced luciferase reporter activity by > 40% when compared to the control (Figure 4F). When the miR-138-5p target sites were mutated in the luciferase reporter, no significant change in luciferase activity was noted following transfection with miR-138-5p and the luciferase reporter (Figure S3). Importantly, a pull-down assay using biotin-coupled miR-138-5p mimic exhibited an obvious enrichment of circSAMD4A compared with the control, while there was no enrichment with the negative control cicrANRIL (Figure 4G). Also, there was a significant negative correlation between circSAMD4A and miR-138-5p expression in the adipose tissue using qRT-PCR (Figure 4H). Thus, our results suggested that circSAMD4A knockdown suppresses preadipocyte differentiation by possibly reducing the functionality of miR-138-5p.

### CircSAMD4A inhibits preadipocyte differentiation via miR-138-5p /EZH2 signaling

**Figure 5:**
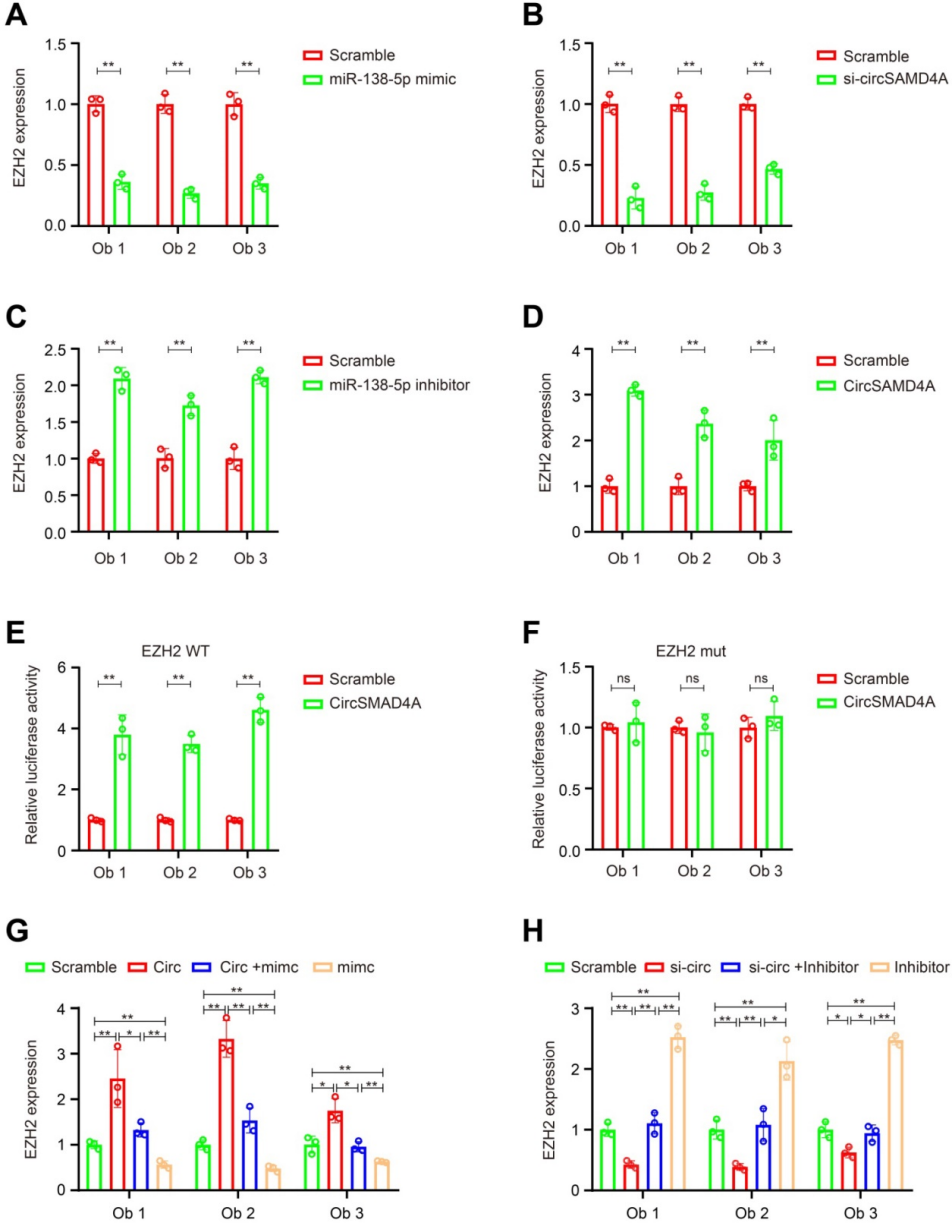
** CircSAMD4A promotes adipogenesis via the miR-138-5p/EZH2 pathway. Quantification of EZH2 expression by qRT-PCR following (A)** miR-138-5p knockdown or** (B)** circSAMD4A overexpression. Quantification of EZH2 expression by qRT-PCR following transfection with **(C)** circSAMD4A-specific siRNAs or **(D)** miR-138-5p mimic was quantified with qRT-PCR. Luciferase assay where preadipocytes were co-transfected with **(E)** scrambled control, circSAMD4A expression plasmid, and a luciferase reporter plasmid containing either wild-type EZH2 (EZH2-WT) or **(F)** EZH2 construct with mutated miR-138-5p binding sites (EZH2-mut). **(G-H)** Reversion assays using vectors overexpressing or konckdown circSAMD4A, as well as miR-138-5p mimics or inhibitors. Data are presented as means ± SD; significant difference was identified with Student's t test. *P < 0.05; **P < 0.01; ns (not significant).

According to previous reports, miR-138-5p can target various proteins associated with adipose differentiation (Figure S4A). Thus, we hypothesized that circSAMD4A induce preadipocyte differentiation by protecting the downregulation of differentiation-promoting factors by miR-138-5p. To test this hypothesis, we overexpressed miR-138-5p mimics, and we measured the expression of its targets by qRT-PCR. Following mimic transfection into preadipocytes, *EZH2* was found to have the most decreased gene expression (Figure 5A, Figure S4B-C and S5A). Furthermore, circSAMD4A knockdown significantly reduced EZH2 expression (Figure 5B and Figure S5C), while its overexpression or miR-138-5p inhibition increased EZH2 expression (Figure 5C-D, Figure S5B and S5D). In a previous study, miR-138-5p could target the 3' UTR of EID-1 27. However, our data showed that miR-138-5p could not inhibit the expression of EID-1 in preadipocytes obviously. Wnt10b and Wnt1, two downstream EZH2 targets 28, were positively correlated with EZH2 changes associated with circSAMD4A and miR-138-5p (Figure S5E-F).

To further examine EZH2, its 3'UTR was cloned into a luciferase vector and the effect of miR-138-5p in the presence or absence of circSAMD4A was investigated. In preadipocytes overexpressing circSAMD4A, the luciferase reporter activity was enhanced in those with the wild-type EZH2 3'UTR when compared to the control, but not in those with a mutated EZH2 3'UTR (Figure 5E-F). However, miR-138-5p upregulation significantly reduced EZH2 expression in the presence of circSAMD4A overexpression (Figure 5G and Figure S6A). Furthermore, miR-138-5p inhibition significantly rescued the downregulation of EZH2 expression after circSAMD4A silencing (Figure 5H and S6B). These data suggested that circSAMD4A induces preadipocyte differentiation by interacting with miR-138-5p within the circSAMD4A/miR-138-5p/EZH2 axis.

To explore the clinical implications of these findings, circSAMD4A and EZH2 expression was examined in VAT from obese patients (n = 60) using immunohistochemistry and ISH. Strong circSAMD4A staining was observed in 58.3% (35/60) of the samples, while 48.3% (29/60) of samples exhibited strong EZH2 expression (Figure S7A). A positive correlation between circSAMD4A and EZH2 expression was further confirmed using qRT-PCR (Figure S7B and Figure S8A-B). Collectively, these findings indicated that circSAMD4A may induce adipocyte differentiation via the miR-138-5p/EZH2 signaling pathway (Figure S7C).

### AAV9-mediated circSAMD4A downregulation corrects obesity

**Figure 6:**
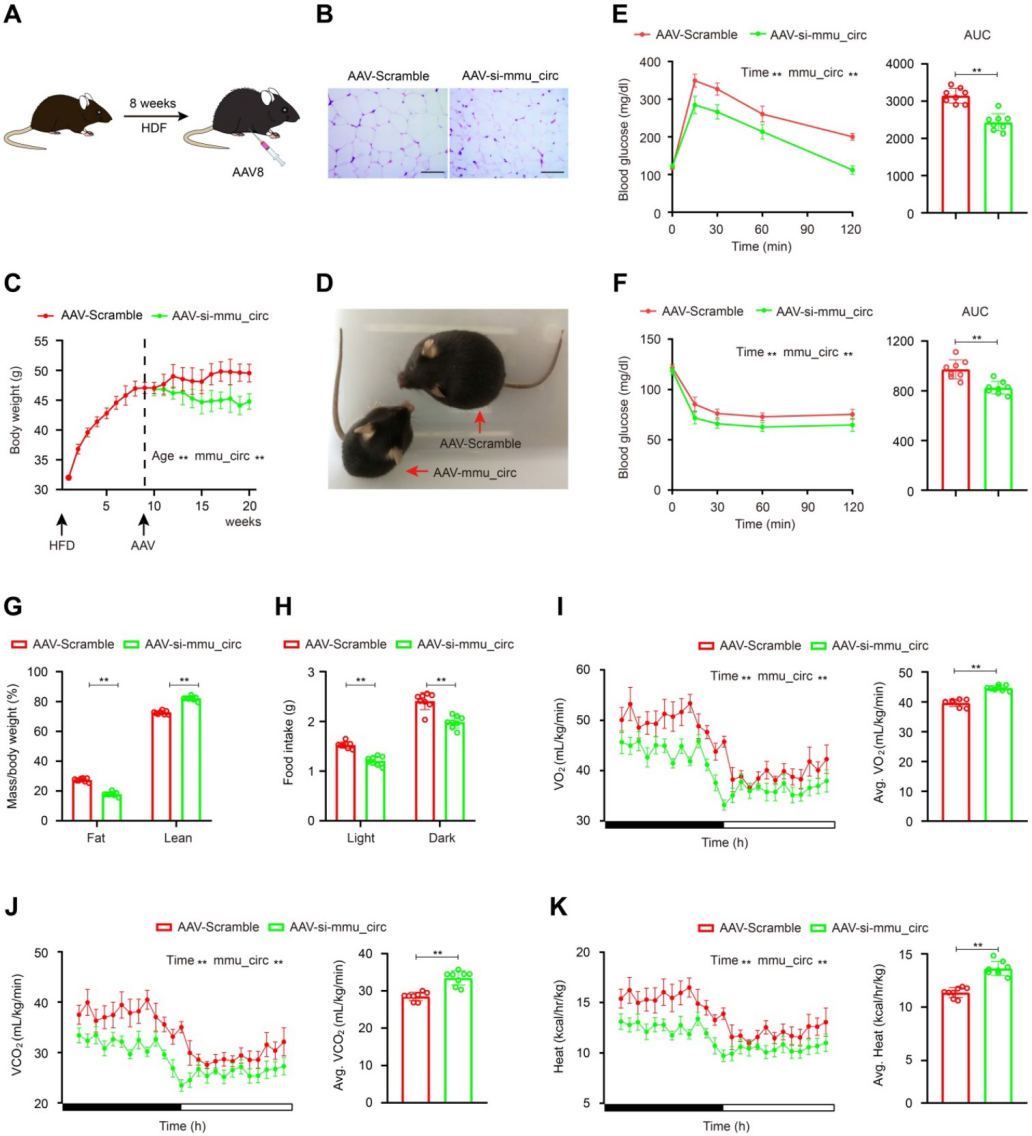
** AAV9-mediated circSAMD4A downregulation counteracts HFD-induced obesity. (A)** Administration of AAV9 vectors through tail vain injection of mice. **(B)** C57BL6 mice were fed HFD for 8 weeks and then administered with AAV-si-mmu_circ0000529 vectors (AAV-si-mmu_circ). Control mice received AAV-Scramble. Representative images of the HE staining of the VAT from mice administered with either AAV vectors.** (C)** Body weight in C57BL6 mice treated with AAV-si-mmu_circ or AAV-Scramble. **(D)** Representative images of the mice administered with AAV-si-mmu_circ or AAV-Scramble. **(E)** Glucose tolerance was determined after the intraperitoneal injection of glucose. **(F)** Insulin sensitivity was determined after an intraperitoneal injection of insulin. **(G)** Fat and lean mass of circRNA knockdown and control mice were determined. **(H)** Food intake, (**I**) O2 (**J**) CO2 production and** (K)** Heat generation consumption were measured during dark and light cycles in a 24-hour feeding period in metabolic chambers. Data are presented as means ± SD; significant difference was identified with Student's t test. *P < 0.05; **P < 0.01. Scale bar = 100µm.

To verify these results *in vivo*, homologous mouse sequences for circSAMD4A were identified using the National Centre for Biotechnology Information Basic Local Alignment Search Tool (BLAST). The circBase database (http://www.circbase.org/) was also consulted and the mouse circular RNA mmu_circ_0000529 was identified that exhibited the best sequence match with circSAMD4A (total score = 750, E-value = 0, and identity = 93%; Figure S9A-B) and was therefore tested in a mouse model. C57BL6 mice (2-month-old) were fed HFD for 10 weeks to obtain obese animals (75% body weight gain), which were then multi-point administered intraperitoneally or subcutaneously with AAV9 vectors encoding a mmu_circ_0000529 siRNA sequence (Figure 6A-B, S9C-D and S10A-B). In the control group, the body weight of AAV9-null mice was unchanged. However, in the AAV-si-mmu_circ_0000529 treated mice, their body weight was corrected after a few weeks (Figure 6C-D). To examine the metabolic effect of mmu_circ_0000529 downregulation, insulin sensitivity and glucose tolerance test were performed that showed increased levels (Figure 6E-F). Also, mice with downregulated mmu_circ_0000529 had significantly less fat mass and more lean mass when compared to the controls (Figure 6G). Besides, mmu_circ_0000529 knockdown mice exhibited a significant decrease in food intake (Figure 6H).

To further characterize metabolic changes associated with mmu_circ_0000529 knockdown, oxygen consumption (VO_2_), carbon dioxide production (VCO_2_), and heat production were examined. In the mmu_circ_0000529 knockdown mice, VO_2_ and VCO_2_ levels were elevated relative to the controls (Figure 6I-J). Also, heat production showed an increase when examined using indirect calorimetry (Figure 6K). Furthermore, EZH2, C/EBP-α and PPAR-γ expression levels were decreased in both VAT and subcutaneous adipose tissue (SAT) samples that were obtained from mice treated with AAV-si- mmu_circ_0000529 (Figure S1C and S10C-E).

## Discussion

Obesity, which poses a serious threat to human health, is becoming increasingly more common worldwide 29. We performed circRNA microarray analysis and identified 244 differentially expressed circRNAs in VAT samples obtained from obese and lean humans (Figure 1). We then focused on the top most upregulated (n=10) and the top most downregulated (n=5) circRNAs (Table S2) and identified circSAMD4A as the most differentially expressed circRNA between the obese and lean VAT samples. CircSAMD4A was significantly upregulated in obese patients compared to lean individuals. It has been reported that circSAMD4A can be detected in human serum (GSE136113), lung fibroblasts 30, foreskin fibroblasts 31, central nervous system (CNS) 32, and adipose tissues 33. We found that circSAMD4A was derived from a hSAMD4A exon and mainly located in the cytoplasm (Figure 2E). Also, circSAMD4A expression was positively correlated with hSAMD4A expression in the obese patients (Figure S8C). Previous data showed that more than 40 circRNA isoforms are produced from the hSAMD4A gene 30. In the present study, we found that 9 circRNA isoforms differential expression between obese patients and lean patients (Table S2), which the expression upregulation of circ0004868 was the most significantly (Figure 1F). Furthermore, circSAMD4A overexpression was found to potentially regulate preadipocytes differentiation and can effectively predict obese human outcomes. We analyzed circRNA differential expression using a microarray platform and detected a much larger number of differentially expressed circRNAs than could be detected by conventional sequencing method 34-36, By using microarray analysis, we identified circSAMD4A upregulation in the adipose of obese patients. Moreover, our study showed that inhibition of mmu_circ_0000529 overexpression in adipose tissues of obese mice could increase insulin sensitivity and glucose tolerance, implicating circSAMD4A in the pathogenesis of diabetes.

Furthermore, we constructed a circRNA-miRNA interaction network to identify the most informative circRNA candidates. It was hypothesized that functionally dysregulated circRNAs could effectively capture target miRNAs and modulate their activity 37. Following miRNA-targeting and cirRIP analyses, our findings suggested that circSAMD4A might act as a miRNA sponge by interacting with miR-138-5p (Figure 4). In preadipocytes, RNA-FISH analysis showed that circSAMD4A and miR-138-5p were co-localized. These findings supported the notion that circRNAs can affect gene expression by acting as “miRNA sponge”. Additionally, while examining the structure of circSAMD4A, we found an internal ribosome entry site (IRES), which can possibly induce 5'-cap independent translation (Figure S11) suggesting its translational potential. While these findings indicated that circSAMD4A acted as a miRNA sponge, other potential functions still require further elucidation.

While circSAMD4A was found to interact with miR-138-5p, the miRNA interacting with circRNAs is not unique and has been associated with glioma angiogenesis 38. Also, in chondrocytes and muscles, circAtp9b has been shown to affect extracellular matrix catabolism and inflammation by directly targeting miR-138-5p 39, while circFoxO3 also targets miR-138-5p to affect differentiation 40. Thus, this new interaction between circSAMD4A and miR-138-5p we identified as well as the observation that circSAMD4A might act as a sponge for miR-138-5p in obesity are interesting and require further investigation.

In a previous study examining human adipose tissue-derived mesenchymal stem cells, miR-138-5p was found to inhibit adipogenic 27. Herein, our observation that differentiation of preadipocytes increased with reduced miR-138-5p is consistent with previous studies 27. Moreover, in preadipocytes overexpressing circSAMD4A, the addition of a mir-138-5p mimic partially reversed the increased differentiation levels (Figure 5G). In previous studies, miR-138-5p has been shown to directly suppress tumour progression by targeting cyclin D3 41 and SOX4 42,43. In another study examining mesenchymal stem cell differentiation into adipocytes, miR-138-5p was shown to inhibit differentiation by targeting the 3' UTR of EID-1 27. However, in our system, miR-138-5p could not inhibit the expression of EID-1 obviously (Figure S4). One possible explanation might be that expression of EID-1 after miR-138-5p transfection was detected in mesenchymal stem cells isolated from SAT, but our study was conducted using the preadipocytes isolated from VAT. Other studies have also shown that miR-138-5p could inhibit the promoter of the epigenetic regulator EZH2 in cancers and nervous system diseases 43-45. Herein, our findings suggest that in obesity, miR-138-5p could interact with the 3'UTR of EZH2, as confirmed by the dual luciferase assay, and also inhibit its expression, thereby suppressing preadipocyte differentiation.

EZH2 is a polycomb-group (PcG) protein 46 and PcG proteins are involved in the suppression of gene transcription 47,48. In one study, its knockdown was shown to inhibit histone H3 lysine 27 (H3K27me3) from associating with the *Wnt* promoter, thereby blocking the gene expression of *Wnt1* and *Wnt10*, and inhibiting adipogenesis 49 In mice with diet-induced obesity, GSK126, an *Ezh2*-specific inhibitor, rescued the obese phenotype by promoting the differentiation of thermogenic beige adipocytes 50. In our study, the ectopic expression of miR-138-5p was blocked by EZH2 3'UTR activity while circSAMD4A overexpression effectively reversed this inhibition. This finding indicated that circSAMD4A could target miR-138-5p and subsequently promote EZH2 in preadipocytes. Moreover, EZH2 protein or mRNA expression was relatively high in adipocytes obtained from obese patients, who had increased cicrSAMD4A expression (Figure S5). Thus, these findings provided evidence for the posttranscriptional regulation of EZH2 by circRNAs in preadipocytes.

Overall, this study identified more than 200 circRNAs with altered expression levels in the adipose tissues derived from obese human patients using circRNA microarray analysis. Of the identified circRNAs, circSAMD4A expression was significantly higher and this increased expression was correlated with a poor post-surgery prognosis in obese patients. Furthermore, the circSAMD4A-miR-138-5p-EZH2 axis was identified and shown to affect preadipocyte differentiation in obese patients. These findings were further confirmed in an obesity mouse model where mmu_circ_0000529 knockdown was demonstrated to rescue the obese phenotype. We also purified exosomes using the culture medium (CM) of preadipocytes and serum samples, and found that both CM and serum exosomal circSAMD4A levels were significantly higher in obese patients than in lean patients (Figure S12). Our results provide a foundation for further functional, diagnostic, and therapeutic studies related to circRNAs in obese humans.

## Supplementary Material

Supplementary figures, tables, and methods.

## Abbreviations

circRNA: circular RNA
HFD: high fat diet
EZH2: enhancer of zeste 2 polycomb repressive complex 2 subunit
ncRNA: non-coding RNA
miRNA: microRNA
lncRNA: long non-coding RNA
RBP: RNA binding protein
Ob: obese
Ln: lean
GEO: Gene Expression Omnibus
VAT: visceral adipose tissues
FCS: fetal calf serum
PBS: phosphate buffered saline
RNA-FISH: RNA fluorescent *in situ* hybridization
siRNA: Small interfering RNA
AUC: area under the curve
GTT,ITT: Glucose and insulin tolerance tests, respectively
TD-NMR: time domain-nuclear magnetic resonance
AGO2: antibodies against argonaute 2
