# Supplementary material for: Circular RNA SAMD4A controls adipogenesis in obesity through the miR-138-5p/EZH2 axis
Source: Theranostics. 2020 Mar 26;10(10):4705–19. doi: 10.7150/thno.42417 (PMC7150479; doi:10.7150/thno.42417)
Supplement: Supplementary file 1 — Supplementary figures, tables, and methods. [file thnov10p4705s1.pdf]

Supplementary Figures

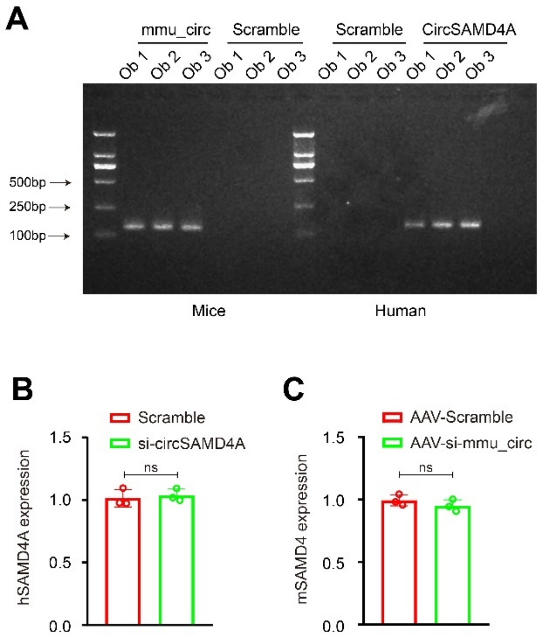

Figure S1 The expression of hSAMD4A and mSAMD4 was detected following circRNA specific knockdown in adipocytes. **(A)** PCR product of circSAMD4A and mmu_circ_0000529 was analyzed using agarose gel electrophoresis. **(B)** The expression of hSAMD4A was detected using qRT-PCR following siRNA transfection in human adipocytes. **(C)** The expression of mSAMD4 was detected using qRT-PCR following AAV9 transfection in mouse adipocytes. Data are presented as means ± SD; significant difference was identified with Student's t test. $*P < 0.05$; $**P < 0.01$; ns (not significant).

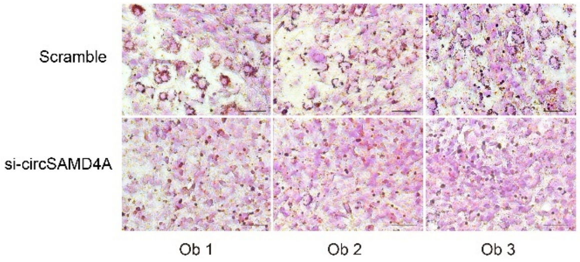

Figure S2 Representative Oil red O staining of differentiated preadipocytes after transfection with circSAMD4A specific siRNA versus scramble controls. Images are representative of three independent experiments. Scale bar = 50μm.

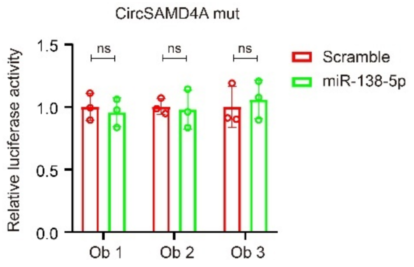

Figure S3 Luciferase assay where preadipocytes were co-transfected with a scrambled control, miR-138-5p mimic, and a luciferase reporter plasmid containing a circSAMD4A construct with mutated miR-138-5p binding sites (circSAMD4A-mut). Data are presented as means ± SD; significant difference was identified with Student's t test. *P < 0.05; **P < 0.01; ns (not significant).

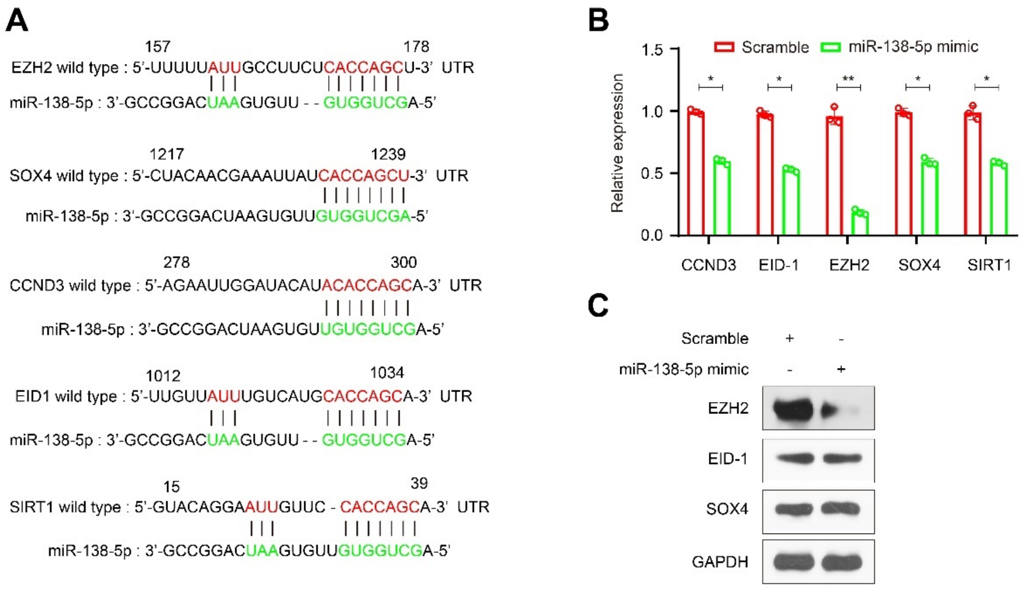

Figure S4 The predicted target molecular of miR-138-5p. **(A)** Schematic of the predicted miR-138-5p sites in the 3'-UTR of target mRNAs. **(B)** The predicted target mRNA expression after transfection with miR-138-5p mimics was quantified with qRT-PCR. **(C)** Proteins expression after transfection with miR-138-5p mimics was analyzed using Western blot with qRT-PCR Data are presented as means ± SD; significant difference was identified with Student's t test. *P < 0.05; **P < 0.01; ns (not significant).

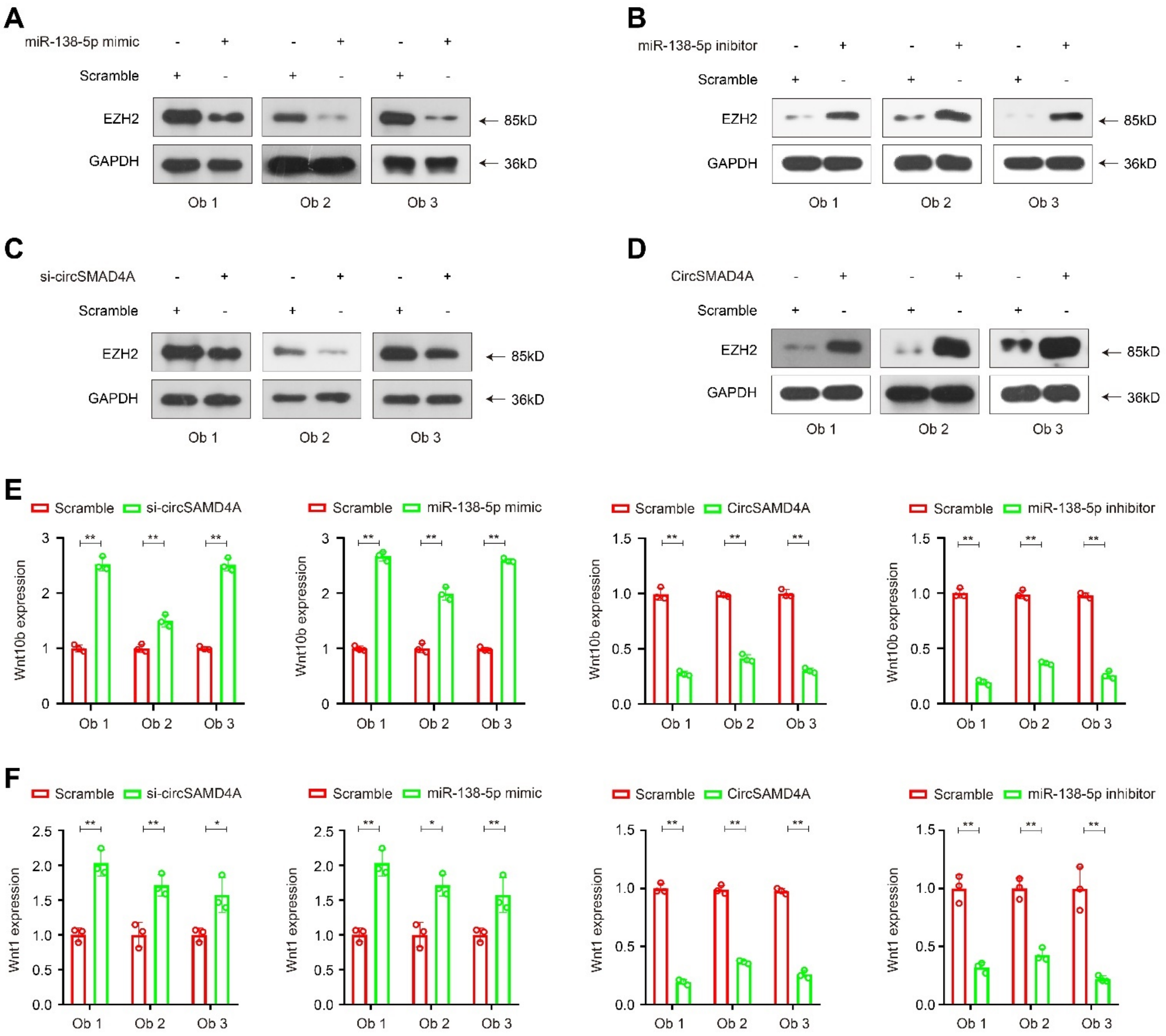
A
miR-138-5p mimic
Scramble
EZH2
GAPDH
85kD
36kD
Ob 1
Ob 2
Ob 3
B
miR-138-5p inibitor
Scramble
EZH2
GAPDH
85kD
36kD
Ob 1
Ob 2
Ob 3
C
si-circSMAD4A
Scramble
EZH2
GAPDH
85kD
36kD
Ob 1
Ob 2
Ob 3
D
CircSMAD4A
Scramble
EZH2
GAPDH
85kD
36kD
Ob 1
Ob 2
Ob 3
E
Scramble
si-circSAMD4A
miR-138-5p mimic
CircSAMD4A
miR-138-5p inhibitor
Wnt10b expression
Ob 1
Ob 2
Ob 3
F
Scramble
si-circSAMD4A
miR-138-5p mimic
CircSAMD4A
miR-138-5p inhibitor
Wnt1 expression
Ob 1
Ob 2
Ob 3

Figure S5 EZH2 and downstream gene expression was influenced by circSAMD4A and miR-138-5p. The EZH2 expression was analyzed using Western blot after **(A)** mir-138-5p mimic or **(B)** inhibitor transfection in adipocytes. EZH2 expression was analyzed using Western blot after circSAMD4A **(C)** siRNA or **(D)** over-expression vector transfection in adipocytes. qRT-PCR assay was performed to analyze the expression of **(E)** Wnt10b and **(F)** Wnt1 after circSAMD4A or miR-138-5p changed in adipocytes from obesity patients. Data are presented as means ± SD; significant difference was identified with Student's t test. *P < 0.05; **P < 0.01; ns (not significant).

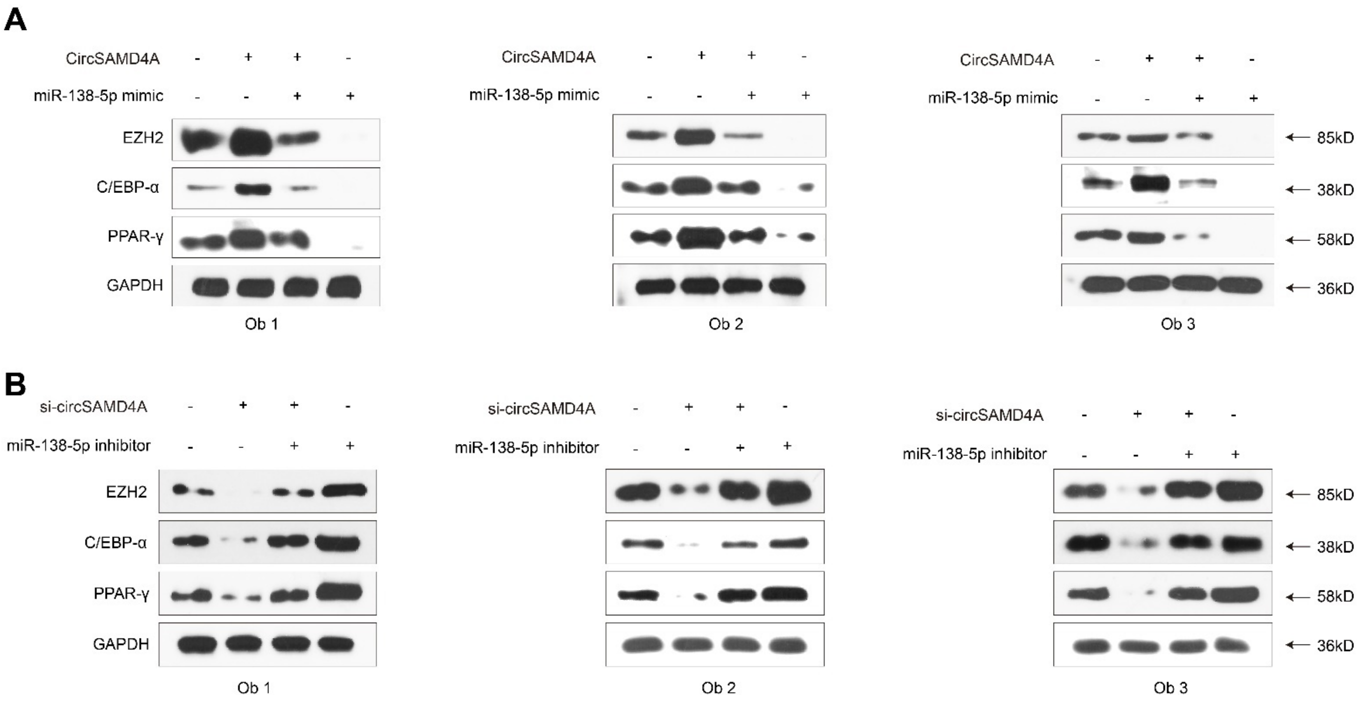

Figure S6 CircSAMD4A counteracted miR-138-5p to promote EZH2 and downstream gene expression. **(A)** The expression of EZH2 was detected using Western blot following circSAMD4A overexpression vector or miR-138-5p mimic transfection in adipocytes from obesity patients. **(B)** The expression of EZH2 was detected using Western blot following circSAMD4A specific siRNA or miR-138-5p inhibitor transfection in adipocytes from obesity patients.

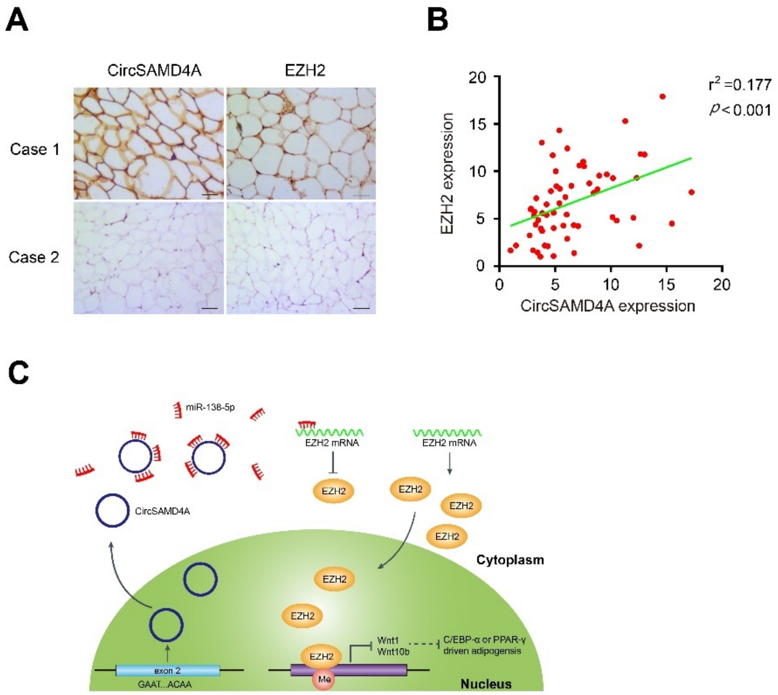

Figure S7 CircSAMD4A was positively correlated with EZH2 expression. **(A)** Representative adipose tissue images showing circSAMD4A in situ hybridization and EZH2 immunohistochemistry. **(B)** Analysis of circSAMD4A (qRT-PCR) and EZH2 (IHC) in adipose tissues, showing linear regressions and significant Pearson correlations. **(C)** CircSAMD4A might induce differentiation via the miR-138-5p/EZH2 signaling pathway.

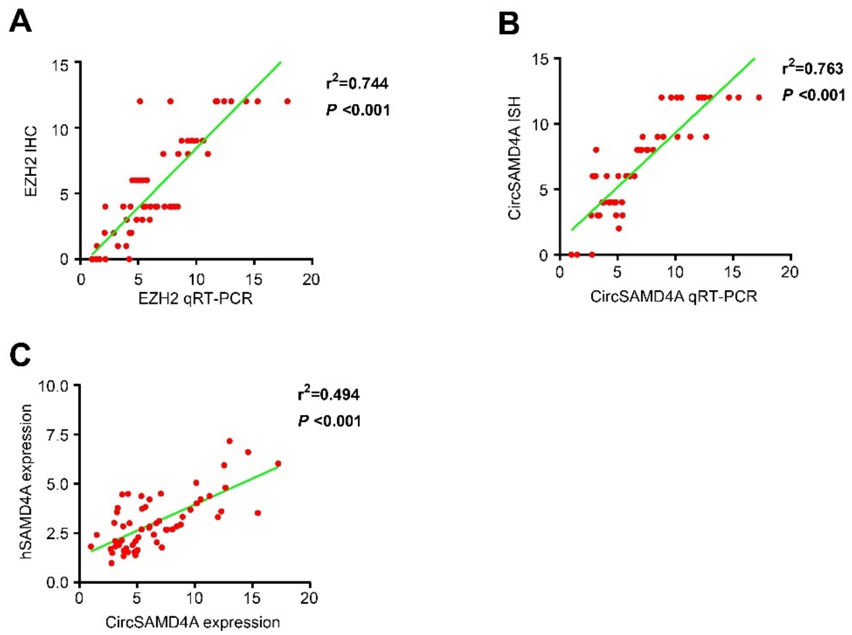

FigureS8 Pearson correlation analysis in adipose tissues. **(A)** Pearson correlation between Immunohistochemistry (IHC) analysis and qRT-PCR analysis of EZH2 in the adipose tissues of 60 obese patients. **(B)** Pearson correlation between ISH analysis and qRT-PCR analysis of circSAMD4A in the adipose tissues of 60 obese patients. **(C)** Pearson correlation between circSAMD4A expression and hSAMD4A expression in the adipose tissues of 60 obese patients. Data are presented as means ± SD; significant difference was identified with Student's t test. *$P < 0.05$; **$P < 0.01$; ns (not significant).

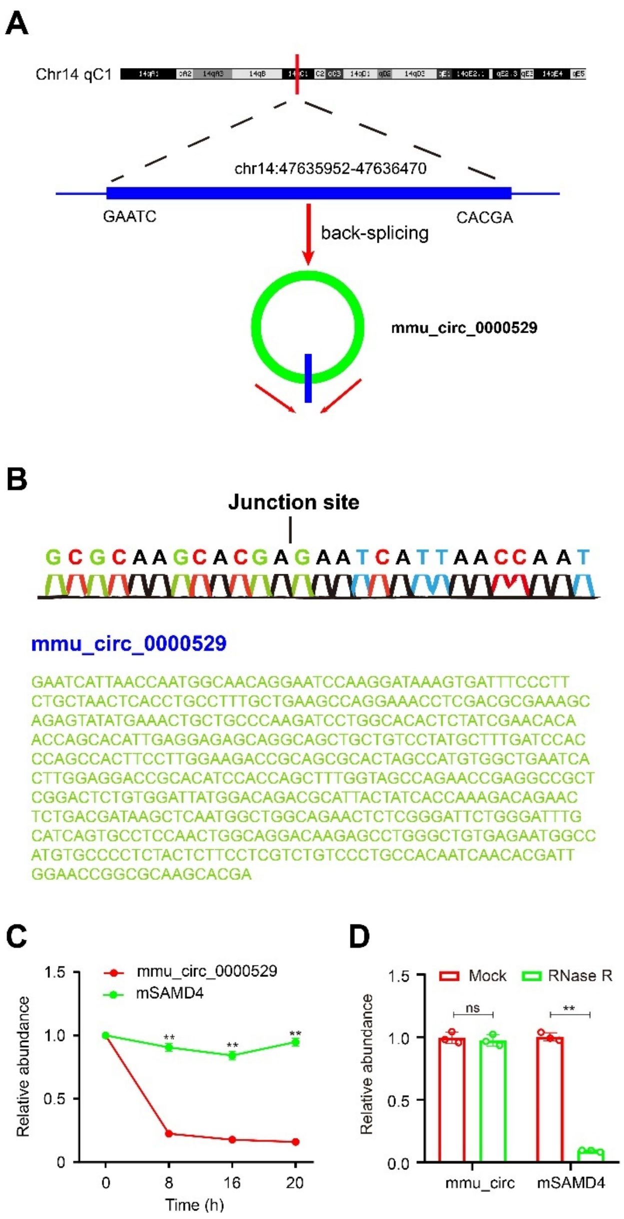
A
Chr14 qC1
chr14:47635952-47636470
GAATC
CACGA
back-splicing
mmu_circ_0000529
B
Junction site
GCGCAAGCACGAGAATCATTAACCAAT
mmu_circ_0000529
GAATCATTAACCAATGGCAACAGGAATCCAAGGATAAAGTGATTTCCCTT
CTGCTAACTCACCTGCCTTTGCTGAAGCCAGGAAACCTCGACGCGAAAGC
AGAGTATATGAAACTGCTGCCCAAGATCCTGGCACACTCTATCGAACACA
ACCAGCACATTGAGGAGAGCAGGCAGCTGCTGTCCTATGCTTTGATCCAC
CCAGCCACTTCCTTGGAAGACCGCAGCGCACTAGCCATGTGGCTGAATCA
CTTGGAGGACCGCACATCCACCAGCTTTGGTAGCCAGAACCGAGGCCGCT
CGGACTCTGTGGATTATGGACAGACGCATTACTATCACCAAAGACAGAAC
TCTGACGATAAGCTCAATGGCTGGCAGAACTCTCGGGATTCTGGGATTTG
CATCAGTGCCTCCAACTGGCAGGACAAGAGCCTGGGCTGTGAGAATGGCC
ATGTGCCCCTCTACTCTTCCTCGTCTGTCCCTGCCACAATCAACACGATT
GGAACCGGCGCAAGCACGA
C
mmu_circ_0000529
mSAMD4
Relative abundance
1.5
1.0
0.5
0
**
**
**
0
8
16
20
Time (h)
D
Mock
RNase R
Relative abundance
1.5
1.0
0.5
0.0
ns
**
mmu_circ
mSAMD4

Figure S9 Characterization of mmu_circ_0000529 in preadipocytes. **(A)** The genomic location of the mSAMD4 gene and of mmu_circ_0000529. **(B)** Sanger sequencing showing the “head-to-tail” splicing of mmu_circ_0000529 in the preadipocytes. **(C)** qRT-PCR quantification of mmu_circ_0000529 and mSAMD4 mRNA expression in preadipocytes after treatment with RNase R. **(D)** qRT-PCR quantification of mmu_circ_0000529 and mSAMD4 mRNA expression in preadipocytes after treatment with Actinomycin D. Data are presented as means ± SD; significant difference was identified with Student's t test. $*P < 0.05$; $**P < 0.01$; ns (not significant).

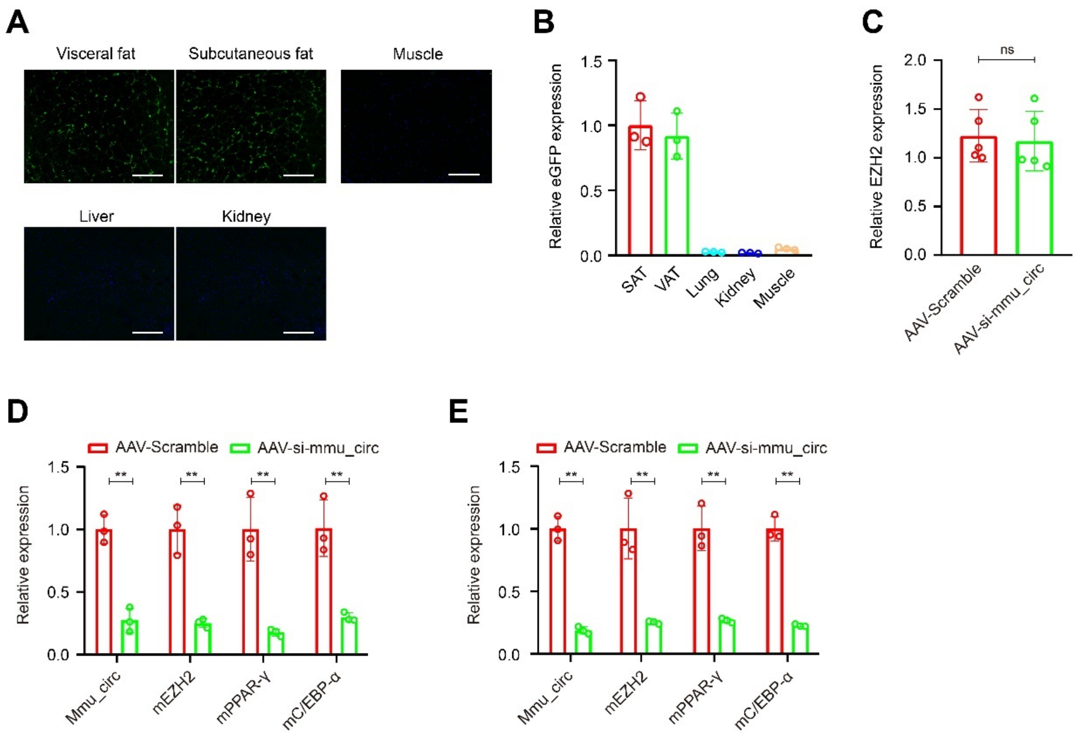

Figure S10 AAV9 administration targeted adipocytes and inhibited preadipocytes differentiation gene expression. **(A)** eGFP expression was observed in SAT, VAT, liver and kidney tissues of obese mice 14 days after AAV9 administration. **(B)** Relative expression of eGFP was detected in adipocytes and off-target control tissue. **(C)** Relative EZH2 expression in muscle of mice was analyzed after vector administration. mmu_circ_000052, mEZH2, mC/EBP-α and mPPAR-γ expression were analyzed in **(D)** subcutaneous fat (SAT) and **(E)** visceral adipose tissue (VAT) of mice after vector administration. Data are presented as means ± SD; significant difference was identified with Student's t test. $*P < 0.05$; $**P < 0.01$; ns (not significant). Scale bar = 250μm.

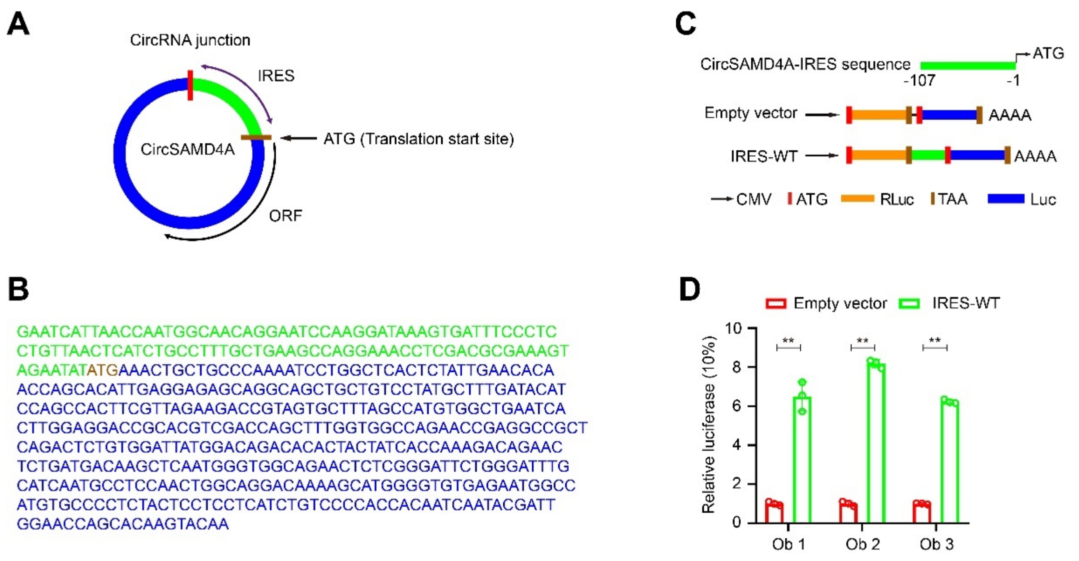

Figure S11 Evaluation of the coding ability of circSAMD4A. **(A)** The putative open reading frame (ORF) in circSAMD4A. **(B)** The sequences of the putative ORF are shown in blue, internal ribosomal entrance site (IRES) sequences are shown in green. **(C)** IRES sequences in circSAMD4A were cloned between Rluc and Luc reporter genes with independent start and stop codons. **(D)** The relative luciferase activity of Luc/ Rluc in the above vectors was tested. Data are presented as means ± SD; significant difference was identified with Student's t test. *P < 0.05; **P < 0.01; ns (not significant).

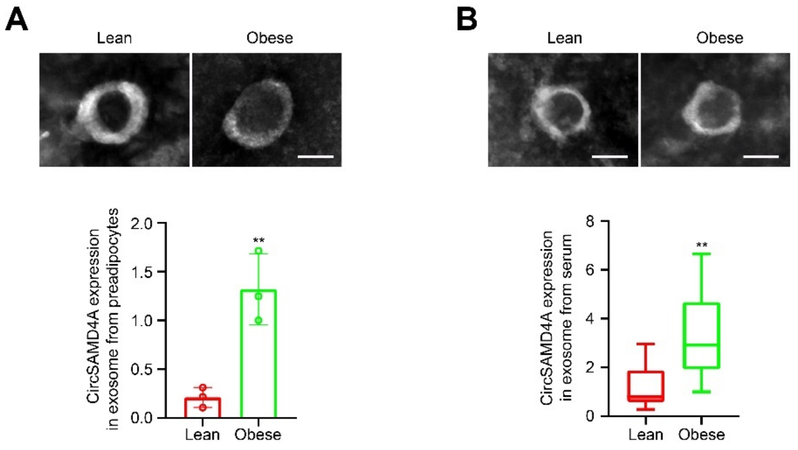

Figure S12 CircSAMD4A expression in exosome from culture medium of preadipocytes and serum. **(A)** Upper: The transmission electron micrograph showed exosomes from preadipocytes of obese and lean patients. Scale bar=50nm. Lower: The expression of circSAMD4A was detected in exosomes from preadipocytes of obese and lean patients using qRT-PCR. **(B)** Upper: The transmission electron micrograph showed exosomes from serum of obese and lean patients. Lower: The expression of circSAMD4A was detected in exosomes from serum of obese and lean patients using qRT-PCR. Scale bar=50nm. Data are presented as means ± SD; significant difference was identified with Student's t test. *P < 0.05; **P < 0.01; ns (not significant).

Supplementary Tables

Table S1 Obese and lean patients used in the study

	Name	Gender[a]	Age (year)	Diabetes[b]	Hyperuricemia[c] (HUA)	Hyperlipemia[d] (HLP)	Obstructive sleep apnea syndrome[e](OSAS)	Hyperinsulinemia[f] (HINS)	Systolic BP (mm Hg)	Diastolic BP (mm Hg)	Preoperative weight(kg)	Height（cm）	Postoperative weight（kg）
Obese	No 1	0	37	0	0	1	1	1	130	82	70	156	62
	No 2	1	33	1	0	1	1	0	160	105	130	188	103
	No 3	1	30	1	0	1	1	0	146	80	123	179	95
	No 4	1	23	0	1	1	1	0	125	85	121	175	90
	No 5	1	31	0	0	1	1	0	146	86	120	174	71.4
	No 6	1	34	0	0	1	1	1	134	88	97.3	167	75
	No 7	1	28	0	0	1	1	0	129	80	129	172	77.5
	No 8	1	19	0	1	0	1	0	131	79	130	171	75
	No 9	1	22	0	0	1	1	0	135	70	120	180	95
	No 10	1	36	0	0	1	1	0	128	74	141.5	180	75
	No 11	0	22	0	0	1	0	0	140	67	105	163	80
	No 12	0	24	0	0	0	1	0	138	76	145	172	80
	No 13	0	20	0	0	0	0	0	127	81	93	159	55
	No 14	0	22	0	0	1	1	0	112	68	118.5	162	50
	No 15	0	22	0	1	1	1	0	112	74	80	162	72
	No 16	1	25	0	1	1	1	0	120	78	135	193	102
	No 17	0	22	0	0	0	0	0	140	80	152	176	62.5

No 18	0	24	0	1	0	1	0	150	86	150	162	80
No 19	0	24	1	1	1	1	0	122	74	123	161	76.3
No 20	0	23	0	0	1	1	0	136	86	115	160	67.9
No 21	0	32	0	0	1	1	0	134	76	91	155	65
No 22	0	30	0	0	1	0	0	111	76	72	155	61
No 23	0	44	0	0	1	1	0	138	90	73	158	62
No 24	0	26	0	0	0	1	0	121	70	120	168	92.3
No 25	0	43	0	0	1	1	0	125	84	84	157	68
No 26	0	19	1	0	0	0	0	155	114	103	164	65
No 27	0	30	0	0	0	0	0	112	69	84	164	72
No 28	0	33	0	0	1	0	0	100	70	92	162	74
No 29	0	31	0	1	1	0	1	128	74	69	153	59
No 30	0	31	0	0	0	0	0	152	106	75.5	153	64
No 31	0	26	0	0	0	0	0	124	74	79	159	64.8
No 32	0	28	0	0	0	1	0	136	96	95	161	72.7
No 33	0	35	0	0	0	1	0	144	87	94	160	55
No 34	0	28	0	0	1	1	0	110	78	74	157	62
No 35	0	21	0	0	0	0	0	120	78	105	168	80.7
No 36	0	33	0	0	1	1	0	116	74	100	162	73.7
No 37	0	39	0	0	1	0	0	115	75	71	147	58
No 38	0	28	0	0	1	1	0	125	94	114	178	90
No 39	0	27	0	0	0	0	0	124	88	118.5	162	60
No	0	35	1	0	1	0	1	152	105	80.5	165	92.9

	40												
	No 41	0	37	1	1	1	0	1	140	74	117	164	65
	No 42	0	29	1	0	0	1	1	120	70	96.5	160	75
	No 43	0	28	0	0	1	0	0	112	63	75	158	64
	No 44	0	23	0	0	1	1	0	131	85	104	162	80
	No 45	0	26	1	0	1	1	0	129	83	95	163	73
	No 46	0	27	0	0	0	1	0	126	74	108	163	52.5
	No 47	0	34	0	0	1	0	1	116	79	100	163	59.1
	No 48	0	24	0	0	0	0	0	102	63	110	170	62
	No 49	0	31	0	1	0	1	0	127	95	89	154	58
	No 50	0	29	0	0	0	0	0	120	70	89	160	74
	No 51	0	24	0	0	0	0	0	126	85	123	164	72.2
	No 52	0	24	0	0	1	1	0	120	74	111	165	60
	No 53	0	23	0	1	1	1	0	117	80	87	162	72
	No 54	0	28	0	0	0	1	0	139	84	110	160	84
	No 55	0	24	1	0	0	1	0	129	76	101	174	84
	No 56	0	24	0	0	1	1	0	118	89	72.5	155	59.7
	No 57	1	28	0	0	0	0	0	182	90	103	165	60
	No 58	1	28	0	1	0	1	0	142	105	100	167	58.8
	No 59	1	26	0	1	0	1	0	136	72	139	169	76
	No 60	1	25	0	0	1	1	0	151	91	139	166	72
Lean	No 1	0	34	0	0	0	0	0	109	79	65.5	163	
	No 2	0	31	0	0	0	0	0	119	80	75.6	182	

No 3	1	36	0	0	0	0	0	125	74	70	172
No 4	0	34	0	0	0	0	0	128	74	59	164
No 5	0	40	0	0	0	0	0	143	79	60	161
No 6	0	36	0	0	0	0	0	110	70	74	182
No 7	0	45	1	0	1	0	0	142	95	84	188
No 8	0	24	0	0	0	0	0	110	63	64.5	178
No 9	0	35	0	0	0	0	0	121	73	73	178
No 10	1	31	0	0	0	0	0	121	80	73	175
No 11	0	33	0	0	0	0	0	106	74	62	160
No 12	0	34	0	0	0	0	0	108	70	76.5	184
No 13	0	29	0	0	0	0	0	105	78	69	169
No 14	1	26	0	0	0	0	0	121	78	69.5	174
No 15	1	30	0	0	0	0	0	125	82	71	172
No 16	1	41	0	0	1	0	0	129	92	68	175
No 17	1	38	0	0	0	0	0	127	78	64	170
No 18	0	33	0	0	0	0	0	118	80	71	169
No 19	0	26	0	0	0	0	0	117	80	73.5	180
No 20	0	36	0	0	0	0	0	113	84	52	159
No 21	0	37	0	0	0	0	0	124	83	67.3	177
No 22	1	25	0	0	0	0	0	118	80	83	186
No 23	0	31	0	0	0	0	0	131	84	64	167
No 24	1	31	0	0	0	0	0	102	63	52.5	162
No 25	0	33	0	0	0	0	0	119	70	79	184
No 26	0	44	1	0	1	0	0	126	82	63	163

No 27	0	29	0	0	0	0	0	116	63	73	183
No 28	0	42	0	0	0	0	0	105	85	61	157
No 29	1	34	0	0	0	0	0	117	79	59	164
No 30	0	36	0	0	0	0	0	130	78	69	167
No 31	1	42	1	1	0	0	0	125	70	73	174
No 32	0	38	0	0	0	0	0	115	63	62	175
No 33	1	32	0	0	0	0	0	110	70	59	170
No 34	1	30	0	0	0	0	0	116	74	58	171
No 35	1	34	0	0	0	0	0	125	75	70	177
No 36	1	29	0	0	0	0	0	119	81	65	173
No 37	0	32	0	0	0	0	0	110	70	66.5	170
No 38	1	32	0	0	0	0	0	115	85	73	174
No 39	0	31	0	0	0	0	0	110	82	53	165
No 40	1	27	0	0	0	0	0	118	63	80	180

[a] “0” means female and “1” means male.
[b] “0” means no Diabetes and “1” means having Diabetes.
[c] “0” means no Hyperuricemia and “1” means having Hyperuricemia.
[d] “0” means no Hyperlipemia and “1” means having Hyperlipemia.
[e] “0” means no OSAS and “1” means having OSAS.
[f] “0” means no Hyperinsulinemia and “1” means having Hyperinsulinemia.

Table S2 Differential expression of circRNAs between the obese and lean VAT samples using circRNA microarray

CircRNA_ID	p values	q values	fold change	Obese 1	Obese 2	Obese 3	Lean 1	Lean 2	Lean 3	hostgene
hsa_circ_0031995	0.00000	0.00035	2.3937	5.1742	5.1309	5.1820	3.9189	3.9082	3.8825	SAMD4A
hsa_circ_0035019	0.00006	0.00736	9.6676	7.2752	7.2464	6.9933	3.7654	3.7707	4.1424	CKMT1B
hsa_circ_0004846	0.00023	0.01847	2.2377	6.4782	6.3192	6.2529	5.2882	5.1038	5.1755	SAMD4A

hsa_circ_0002612	0.00043	0.02600	2.4985	6.4262	6.2688	6.1165	4.9429	4.8100	5.0976	SAMD4A
hsa_circ_0002012	0.00056	0.02732	2.0258	4.9984	4.7935	4.7812	3.9414	3.6973	3.8784	SAMD4A
hsa_circ_0024182	0.00118	0.03885	0.3866	1.9604	1.5729	1.6124	3.0065	2.9589	3.3014	ACAT1
hsa_circ_0035016	0.00130	0.03885	4.8295	3.6524	4.3481	4.0803	1.7391	1.4246	2.1046	CKMT1B
hsa_circ_0031990	0.00142	0.03885	2.4310	6.1779	6.3589	6.1107	5.1551	4.8443	4.7873	SAMD4A
hsa_circ_0031996	0.00173	0.03885	2.3434	5.6522	5.3668	5.7719	4.2092	4.5074	4.4022	SAMD4A
hsa_circ_0027396	0.00181	0.03885	2.0440	3.4891	3.5476	3.5912	2.3914	2.4892	2.6435	C12orf66
hsa_circ_0023599	0.00185	0.03885	0.4806	6.1459	5.9340	5.7906	7.1671	7.0517	6.8242	XRRA1
hsa_circ_0083215	0.00191	0.03885	2.0301	7.6295	7.7918	7.9848	6.9429	6.7862	6.6158	ESYT2
hsa_circ_0068162	0.00249	0.03990	3.4653	3.0658	3.7330	3.5018	1.8598	1.3614	1.7326	MCF2L2
hsa_circ_0023595	0.00254	0.03990	0.3860	3.9216	4.2087	3.8286	5.2713	5.4096	5.4209	XRRA1
hsa_circ_0005187	0.00272	0.03990	2.1125	3.6406	3.5233	3.7603	2.4908	2.4010	2.7778	---
hsa_circ_0031993	0.00273	0.03990	2.0457	8.7053	8.3247	8.4303	7.4843	7.2581	7.6239	SAMD4A
hsa_circ_0055859	0.00284	0.03990	0.3847	2.5717	2.7941	3.1134	4.3746	4.3131	3.9407	NPAS2
hsa_circ_0087875	0.00294	0.03990	0.4189	2.0885	1.8995	2.3882	3.2081	3.4156	3.5417	IKBKAP
hsa_circ_0023592	0.00327	0.04090	0.4279	4.2041	4.3600	3.8796	5.1117	5.5454	5.4650	XRRA1
hsa_circ_0031916	0.00352	0.04090	0.4311	3.4776	3.3261	3.8209	4.5986	4.9551	4.7350	FRMD6
hsa_circ_0030322	0.00353	0.04090	0.4541	2.9066	3.2484	2.8910	3.9105	4.3859	4.1568	ATP7B
hsa_circ_0016194	0.00397	0.04090	0.4502	3.1719	2.6972	2.8451	3.8605	4.3088	4.0038	DSTYK
hsa_circ_0023593	0.00417	0.04090	0.3812	3.9036	4.1990	3.6647	5.3253	5.4611	5.1927	XRRA1
hsa_circ_0070784	0.00434	0.04090	2.2568	5.5749	6.0128	6.0340	4.7304	4.5055	4.8830	USP53
hsa_circ_0058251	0.00470	0.04090	0.4172	2.7436	3.0998	2.6462	3.9201	3.9207	4.4145	USP37
hsa_circ_0070817	0.00482	0.04090	0.4899	5.8844	5.7485	5.5303	6.8283	6.9279	6.4798	PRDM5
hsa_circ_0002722	0.00489	0.04090	2.0217	5.0905	4.9613	5.0907	3.9726	4.2305	3.8727	---
hsa_circ_0028003	0.00495	0.04090	0.4588	4.2693	4.7101	4.7264	5.5592	5.9055	5.6345	C12orf75
hsa_circ_0076084	0.00510	0.04090	2.0382	5.9267	5.8949	6.1700	4.9467	4.7365	5.2043	ANKS1A
hsa_circ_0063803	0.00519	0.04090	2.4938	5.6402	6.2545	5.9289	4.8783	4.6359	4.3763	CELSR1
hsa_circ_0031989	0.00520	0.04090	2.2677	6.3203	6.3629	6.2586	4.9589	5.0891	5.3275	SAMD4A
hsa_circ_0045518	0.00565	0.04257	2.1225	4.4444	4.7045	4.8380	3.5036	3.3456	3.8590	ABCA5
hsa_circ_0035024	0.00576	0.04257	8.5228	8.9714	9.0720	8.8145	5.7334	5.3429	6.3413	CKMT1A
hsa_circ_0044242	0.00624	0.04334	0.2959	4.9170	4.4838	5.3174	6.5546	6.9692	6.5426	C17orf57
hsa_circ_0084014	0.00632	0.04334	2.1622	5.2896	5.4019	5.7947	4.4383	4.1106	4.6066	TACC1
hsa_circ_0031915	0.00664	0.04334	0.4138	4.0101	3.8445	4.3661	4.9762	5.5565	5.4899	FRMD6

hsa_circ_0021316	0.00672	0.04334	0.2430	2.2101	2.8353	3.1770	4.8619	5.2196	4.2692	ARNTL
hsa_circ_0056117	0.00698	0.04334	2.0531	5.3466	5.3404	5.0304	4.1168	3.9727	4.4867	MERTK
hsa_circ_0055864	0.00705	0.04334	0.2861	2.8427	2.3753	3.2521	4.6444	5.0538	4.1922	NPAS2
hsa_circ_0011062	0.00710	0.04334	2.2084	7.9048	8.0893	7.6207	7.0446	6.4649	6.6525	MAP3K6
hsa_circ_0024056	0.00749	0.04460	2.1176	3.5481	3.6418	4.0746	2.9041	2.4769	2.6605	CWC15
hsa_circ_0062282	0.00771	0.04477	2.2643	3.5790	3.7214	3.3175	2.3846	2.4051	2.3182	ARVCF
hsa_circ_0056121	0.00821	0.04551	2.0903	6.2065	6.7026	6.2723	5.5210	5.2724	5.2314	MERTK
hsa_circ_0088147	0.00838	0.04551	0.4067	2.4777	2.8829	2.4075	3.9691	3.9130	3.8232	RGS3
hsa_circ_0069162	0.00860	0.04551	3.7628	5.8656	6.8328	6.6087	4.2172	4.3166	5.0530	SH3TC1
hsa_circ_0055866	0.00861	0.04551	0.3640	3.4351	3.3863	3.4607	5.0526	4.9751	4.5872	NPAS2
hsa_circ_0003980	0.00941	0.04551	0.3066	2.1616	1.9429	1.7643	3.7736	3.9616	3.1620	NPAS2
hsa_circ_0052127	0.00944	0.04551	2.1797	3.5590	3.8573	3.8774	2.3446	2.9515	2.5812	NLRP12
hsa_circ_0044007	0.00996	0.04551	2.5799	3.7624	3.4393	4.1577	2.0933	2.3419	2.8167	MPP3
hsa_circ_0032588	0.01031	0.04551	2.1997	4.9041	4.4904	4.4089	3.4759	3.3784	3.5803	LTBP2
hsa_circ_0001172	0.01079	0.04551	0.4860	2.8257	2.7093	2.6272	4.0295	3.5435	3.6739	PPP4R1L
hsa_circ_0028004	0.01087	0.04551	0.4252	6.3246	6.9853	6.8055	7.7062	8.3167	7.7934	C12orf75
hsa_circ_0002579	0.01098	0.04551	2.0111	6.5963	7.1622	6.9579	5.9846	5.6125	6.1065	LDLR
hsa_circ_0026556	0.01110	0.04551	0.3443	1.9543	1.2099	1.7031	2.7125	3.5967	3.1341	SOAT2
hsa_circ_0008132	0.01111	0.04551	0.3048	1.7449	1.1765	2.0812	3.1913	3.0144	3.9150	PRELID2
hsa_circ_0018643	0.01159	0.04551	2.3612	10.2279	10.5703	10.9560	9.3872	9.0450	9.6354	PPA1
hsa_circ_0078452	0.01171	0.04551	2.0396	3.4764	3.4436	3.9191	2.4983	2.5723	2.7249	SYNJ2
hsa_circ_0072525	0.01180	0.04551	0.4626	2.2082	2.2224	2.0153	3.3772	2.9227	3.4388	MAP3K1
hsa_circ_0057361	0.01189	0.04551	0.4523	8.2121	7.5951	7.6268	8.8477	9.2743	8.7823	COL3A1
hsa_circ_0007443	0.01212	0.04551	0.4196	6.2983	5.8354	5.5622	6.9570	7.5366	6.9810	RUNX1T1
hsa_circ_0042349	0.01219	0.04551	0.4249	2.2027	2.1747	1.6868	2.8233	3.5140	3.3968	TRIM16L
hsa_circ_0084879	0.01222	0.04551	0.4430	6.3533	5.7512	5.7341	6.9331	7.4771	6.9763	RUNX1T1
hsa_circ_0055867	0.01235	0.04551	0.3764	2.5531	2.9719	2.1042	4.1009	4.2202	3.5924	NPAS2
hsa_circ_0018647	0.01239	0.04551	2.2435	10.1635	10.3076	10.8166	9.2751	8.9598	9.5746	PPA1
hsa_circ_0057351	0.01278	0.04551	0.4569	6.7638	6.4091	6.3543	7.2574	7.9281	7.6887	COL3A1
hsa_circ_0026442	0.01317	0.04551	5.3309	5.4959	4.6365	4.8290	2.4663	1.7500	3.2590	KRT5
hsa_circ_0031913	0.01332	0.04551	0.4088	5.0676	5.0576	5.7561	6.2076	6.7834	6.8032	FRMD6
hsa_circ_0085781	0.01346	0.04551	2.1506	5.5958	5.4904	5.6262	4.4405	4.1909	4.7214	DENND3
hsa_circ_0025013	0.01353	0.04551	0.4558	4.8127	4.1958	4.1897	5.5579	5.7843	5.3100	DCP1B

hsa_circ_0069157	0.01364	0.04551	2.1901	4.1817	4.4422	4.4187	2.8265	3.3750	3.3937	SH3TC1
hsa_circ_0050060	0.01386	0.04551	2.1260	5.4684	4.9933	5.3032	4.5292	4.0799	3.8459	ARRDC2
hsa_circ_0023991	0.01396	0.04551	0.3025	4.4892	4.9828	4.7544	6.4724	6.8956	5.9100	NAALAD2
hsa_circ_0007973	0.01409	0.04551	3.1412	5.8398	5.7880	6.6894	4.8623	4.2613	4.3514	SMAP2
hsa_circ_0002896	0.01412	0.04551	0.4252	2.6286	2.9160	2.4100	4.1157	4.0627	3.4265	---
hsa_circ_0013802	0.01417	0.04551	0.3762	3.7183	3.0875	3.7732	4.5848	5.4108	4.7755	TCONS_l2_00002625
hsa_circ_0076154	0.01421	0.04551	8.7650	4.4638	6.1513	4.5844	2.9784	1.3776	1.5280	FKBP5
hsa_circ_0074097	0.01449	0.04551	2.1693	4.4820	4.2828	4.8643	3.5380	3.0188	3.6983	GFRA3
hsa_circ_0044236	0.01457	0.04551	2.8473	3.4715	4.1517	4.2606	2.9457	2.3566	2.0159	MYL4
hsa_circ_0026806	0.01487	0.04551	0.2526	11.7001	11.5997	11.7353	13.4007	14.1238	13.3285	RPS26
hsa_circ_0056119	0.01515	0.04551	2.4567	6.0619	6.5795	6.0761	5.0072	4.9069	4.9739	MERTK
hsa_circ_0090323	0.01519	0.04551	0.4901	4.4196	4.7622	4.1265	5.5573	5.5926	5.2978	KDM6A
hsa_circ_0018333	0.01567	0.04551	2.4590	4.4702	5.2774	4.9894	3.7974	3.8696	3.1991	ERCC6
hsa_circ_0018645	0.01603	0.04551	2.2221	10.1577	10.2705	10.8556	9.3166	8.9939	9.5629	PPA1
hsa_circ_0056124	0.01623	0.04551	2.0560	4.9817	5.5873	5.2016	4.0953	4.3693	4.2395	MERTK
hsa_circ_0057733	0.01669	0.04551	0.4083	2.3348	3.0648	3.0604	3.9267	4.3689	4.1221	ORC2
hsa_circ_0009010	0.01680	0.04551	0.4563	5.8007	5.4797	5.5096	7.0871	6.3965	6.6381	PRH1-PRR4
hsa_circ_0049920	0.01705	0.04551	2.0595	4.4166	4.5011	4.4471	3.1792	3.6813	3.3318	MYO9B
hsa_circ_0018648	0.01724	0.04551	2.4915	10.7220	10.6593	11.4446	10.0074	9.5993	9.3237	PPA1
hsa_circ_0007913	0.01728	0.04551	0.4372	3.4187	3.3883	2.8469	4.3720	4.3953	4.5304	WLS
hsa_circ_0006488	0.01794	0.04551	0.4528	4.9818	5.1659	4.7523	6.4776	5.7377	6.0474	TAS2R30
hsa_circ_0003301	0.01818	0.04551	2.9079	3.1521	3.5451	4.1791	1.9401	2.5313	1.8806	SAMD4A
hsa_circ_0029267	0.01837	0.04551	0.4630	3.9734	4.2328	3.5633	4.6144	5.1821	5.2952	TCTN2
hsa_circ_0064888	0.01840	0.04551	0.4709	4.3258	4.3733	3.7997	5.4176	5.4912	4.8304	EXOG
hsa_circ_0018581	0.01841	0.04551	2.7507	3.2223	3.8263	4.1939	1.8827	2.7031	2.3230	DDX21
hsa_circ_0025461	0.01858	0.04551	0.4561	7.2795	6.6355	6.5602	8.2540	7.9008	7.7891	PRH1-PRR4
hsa_circ_0018649	0.01859	0.04551	2.1739	10.4524	10.4737	11.0818	9.7335	9.3827	9.6015	PPA1
hsa_circ_0082718	0.01876	0.04551	0.4869	3.7795	3.3607	3.0577	4.6757	4.4612	4.2340	DENND2A
hsa_circ_0018651	0.01903	0.04551	2.1656	10.3667	10.5324	11.0916	9.6208	9.2251	9.8384	PPA1
hsa_circ_0081898	0.01917	0.04551	0.4948	4.7930	4.1241	4.5935	5.1924	5.8069	5.5706	DUS4L
hsa_circ_0090608	0.01931	0.04551	2.2668	3.4640	3.7491	3.2811	2.1403	2.7566	1.9712	TSPYL2
hsa_circ_0018640	0.01931	0.04551	2.4188	11.0070	10.8541	11.6748	10.2587	9.8196	9.7126	PPA1
hsa_circ_0018639	0.01965	0.04551	2.3662	11.3479	11.1884	11.9771	10.3437	10.0331	10.4963	PPA1

hsa_circ_0013391	0.01967	0.04551	0.2715	1.0508	1.7270	2.0379	3.6187	3.5488	3.4538	GPSM2
hsa_circ_0012878	0.01982	0.04551	0.4657	3.2870	2.8956	2.5891	4.3100	4.1539	3.6110	TCONS_l2_00002523
hsa_circ_0018644	0.01985	0.04551	2.4379	10.7827	10.6065	11.4438	10.0574	9.6456	9.3180	PPA1
hsa_circ_0026450	0.02053	0.04551	3.5611	5.6039	4.8483	4.8920	2.7079	2.9570	3.9806	KRT5
hsa_circ_0018638	0.02119	0.04551	2.2725	12.1876	12.1306	12.8410	11.4215	11.0336	11.2381	PPA1
hsa_circ_0076151	0.02152	0.04551	7.9177	7.5620	9.4441	7.9702	6.2971	4.3360	5.4815	FKBP5
hsa_circ_0048170	0.02162	0.04551	3.0707	2.8634	3.1594	3.3330	2.0560	1.2850	0.9753	POLR2E
hsa_circ_0055862	0.02171	0.04551	0.3812	3.1898	3.8361	2.9512	4.9770	5.0084	4.1702	NPAS2
hsa_circ_0049400	0.02177	0.04551	2.8501	6.5037	7.5142	7.2266	5.1841	5.4097	6.1224	LDLR
hsa_circ_0018646	0.02187	0.04551	2.7487	4.3206	4.0787	4.9309	2.9605	3.1907	2.9259	PPA1
hsa_circ_0055861	0.02194	0.04551	0.3847	1.5600	2.4639	2.4370	3.7031	3.8123	3.1723	NPAS2
hsa_circ_0091204	0.02197	0.04551	0.4868	2.9563	2.2060	2.5356	3.4187	3.8237	3.6418	KLHL4
hsa_circ_0070769	0.02204	0.04551	2.0616	7.8898	8.5475	8.3283	7.3591	6.7881	7.4737	USP53
hsa_circ_0092261	0.02221	0.04551	2.0410	8.3319	8.0888	8.7620	7.1172	7.1991	7.7696	DDX3Y
hsa_circ_0074393	0.02250	0.04551	2.7274	3.3548	3.1668	3.1810	1.9824	2.0169	1.2451	SH3RF2
hsa_circ_0026807	0.02263	0.04551	0.2875	10.6396	10.4167	10.4640	12.0581	12.8214	11.8584	RPS26
hsa_circ_0007481	0.02264	0.04551	0.4253	6.6038	6.0264	5.8192	7.5826	7.3527	7.3182	PRH1-PRR4
hsa_circ_0018652	0.02293	0.04551	2.3846	10.4487	10.3217	11.1656	9.7729	9.3878	9.0807	PPA1
hsa_circ_0031904	0.02301	0.04551	0.4009	5.4262	4.9603	5.7842	6.1615	6.9014	7.0363	FRMD6
hsa_circ_0020560	0.02330	0.04551	2.6610	3.1201	3.0306	3.9453	1.9529	1.7199	2.3110	ADAM8
hsa_circ_0070785	0.02384	0.04551	2.2712	6.7336	7.5207	7.3972	6.0796	5.6351	6.4026	USP53
hsa_circ_0005681	0.02392	0.04551	2.2094	5.1348	4.4519	5.2209	4.1083	3.7632	3.5684	MEN1
hsa_circ_0016697	0.02409	0.04551	2.2326	5.2204	5.9932	5.6995	4.2426	4.2203	4.9512	C1orf55
hsa_circ_0018642	0.02416	0.04551	2.2112	10.3076	10.4098	11.0597	9.6745	9.2446	9.5116	PPA1
hsa_circ_0056554	0.02423	0.04551	0.4873	2.9097	2.6663	2.2719	3.6720	3.5993	3.7540	ZRANB3
hsa_circ_0049398	0.02443	0.04551	3.6733	4.7015	5.9331	5.4928	2.8038	3.4377	4.1824	LDLR
hsa_circ_0013803	0.02448	0.04551	0.2921	4.0725	2.6871	3.4449	4.8186	5.7543	5.1189	TCONS_l2_00002625
hsa_circ_0056118	0.02466	0.04551	2.4749	5.0756	5.9413	5.3264	4.3170	3.9579	4.2626	MERTK
hsa_circ_0021577	0.02491	0.04551	0.4902	4.6506	4.0684	4.8221	5.5818	5.7853	5.3259	QSER1
hsa_circ_0026455	0.02502	0.04551	4.8439	5.6832	4.7236	5.0883	2.5780	1.9593	3.7253	KRT5
hsa_circ_0057362	0.02511	0.04551	0.3426	7.7361	6.5259	7.0469	8.5583	9.1257	8.4163	COL3A1
hsa_circ_0026413	0.02515	0.04551	4.0030	4.0122	3.5051	3.3749	2.2141	0.7329	1.6585	KRT6C
hsa_circ_0074362	0.02518	0.04551	2.1265	5.4297	6.1987	6.0443	5.1349	4.4791	4.8282	ARHGAP26

hsa_circ_0019498	0.02583	0.04596	2.2738	3.7325	3.7762	3.8033	2.2015	2.5671	2.9040	CHUK
hsa_circ_0086312	0.02600	0.04596	2.1693	3.3200	2.9953	3.0257	1.9765	2.3716	1.5475	GLDC
hsa_circ_0004394	0.02614	0.04596	0.4076	5.5495	4.5988	4.9332	6.3238	6.5954	6.1798	RUNX1T1
hsa_circ_0089758	0.02619	0.04596	2.0013	2.7647	3.2813	3.5106	2.2261	1.9210	2.4550	CACNA1B
hsa_circ_0044243	0.02637	0.04596	0.4327	4.7397	4.2363	5.1459	5.7113	6.0807	6.0671	C17orf57
hsa_circ_0018641	0.02658	0.04599	2.2856	10.6939	10.5939	11.4000	10.1220	9.6975	9.3189	PPA1
hsa_circ_0031912	0.02801	0.04731	0.4197	5.4359	5.0054	5.9558	6.2406	7.0867	6.8586	FRMD6
hsa_circ_0031905	0.02807	0.04731	0.4528	6.2949	6.2059	6.9246	7.1211	7.7991	7.9235	FRMD6
hsa_circ_0045854	0.02829	0.04731	2.0475	7.2736	7.1225	7.7639	6.3661	5.9212	6.7374	SEC14L1
hsa_circ_0042077	0.02848	0.04731	0.2369	1.9957	0.5483	1.6734	3.4439	3.4466	3.8759	GLP2R
hsa_circ_0079215	0.02860	0.04731	0.4387	3.5488	4.4878	4.0661	5.1468	5.5264	5.1082	SDK1
hsa_circ_0004452	0.02878	0.04731	2.0150	6.7797	7.1911	7.4018	5.7825	5.9474	6.5580	---
hsa_circ_0078335	0.02888	0.04731	2.1865	4.3097	4.4200	5.1187	3.4161	3.8215	3.3130	IPCEF1
hsa_circ_0004432	0.02894	0.04731	2.1341	4.9993	5.4483	5.8601	4.6885	4.3228	4.0761	MYO1E
hsa_circ_0057354	0.02997	0.04731	0.4967	7.0294	6.8241	6.8417	7.4812	8.1190	8.0518	COL3A1
hsa_circ_0018454	0.03021	0.04731	2.0950	3.7709	4.4119	4.3749	3.3754	2.6133	3.3339	ARID5B
hsa_circ_0002697	0.03023	0.04731	2.0548	8.0932	7.3684	8.0018	6.5856	6.5415	7.2179	MTHFD1L
hsa_circ_0034055	0.03041	0.04731	0.4993	3.1489	3.2083	2.8473	3.6045	4.3175	4.2163	TUBGCP5
hsa_circ_0075466	0.03079	0.04731	2.0365	5.0162	5.2142	5.7941	4.5566	3.9358	4.4938	SERPINB9
hsa_circ_0044241	0.03109	0.04731	0.3793	4.2810	3.6657	4.8136	5.3985	5.9267	5.8032	C17orf57
hsa_circ_0004895	0.03126	0.04731	2.1540	5.8573	6.2002	6.7412	5.4742	5.2002	4.8824	SYNC
hsa_circ_0019453	0.03132	0.04731	2.1118	4.3596	4.5864	4.4168	3.6921	3.4265	2.9136	ABCC2
hsa_circ_0057355	0.03133	0.04731	0.4831	6.8448	6.6615	6.5420	7.2605	7.9669	7.8879	COL3A1
hsa_circ_0018650	0.03136	0.04731	2.2956	11.3330	11.3000	12.0984	10.6508	10.2873	10.3163	PPA1
hsa_circ_0013694	0.03145	0.04731	2.2308	4.3253	5.1797	4.5893	3.7981	3.5054	3.4273	ATP1A1
hsa_circ_0070762	0.03174	0.04731	2.1102	11.8763	12.5032	12.3326	11.1994	10.6530	11.5580	USP53
hsa_circ_0069158	0.03183	0.04731	2.1756	4.3064	4.7659	4.7677	2.9150	3.7571	3.7067	SH3TC1
hsa_circ_0005044	0.03186	0.04731	0.3791	2.5458	2.7280	3.0749	4.0399	4.7107	3.6411	PRR5L
hsa_circ_0009176	0.03187	0.04731	2.3618	7.0490	7.3596	7.4763	5.6333	5.8024	6.5784	ELL2
hsa_circ_0085785	0.03216	0.04731	2.0726	5.0472	4.5998	5.4524	4.0636	3.7156	4.2418	DENND3
hsa_circ_0061270	0.03272	0.04731	3.6346	6.7851	6.1331	7.3713	4.8505	4.9682	5.1315	SAMSN1
hsa_circ_0074365	0.03294	0.04731	2.0476	7.7780	8.5658	8.1004	7.2990	6.9612	7.1719	ARHGAP26
hsa_circ_0031910	0.03294	0.04731	0.4724	4.4862	4.2562	5.0652	5.2138	5.8709	5.9801	FRMD6

hsa_circ_0074150	0.03304	0.04731	2.0440	4.1976	3.8286	3.8940	2.6314	2.6902	3.3992	HSPA9
hsa_circ_0052828	0.03318	0.04731	0.2749	4.8628	4.3549	4.5846	6.7620	6.7909	5.5942	SDC1
hsa_circ_0045856	0.03378	0.04731	2.0378	7.4373	7.2353	7.7254	6.5422	5.9179	6.7708	SEC14L1
hsa_circ_0074368	0.03398	0.04731	2.0285	4.9920	5.2990	5.1463	4.0706	3.6947	4.5113	ARHGAP26
hsa_circ_0072555	0.03405	0.04731	0.4663	5.8056	5.6777	6.2768	6.7283	6.7327	7.5208	PLK2
hsa_circ_0055135	0.03423	0.04731	0.4822	3.0773	2.8895	3.7037	4.3421	4.4610	4.1366	ANKRD53
hsa_circ_0029168	0.03426	0.04731	2.2014	3.4743	4.0455	4.2756	2.4406	2.5713	3.3184	PITPNM2
hsa_circ_0001600	0.03450	0.04731	9.1125	8.3844	9.3933	8.0159	4.1707	4.0841	6.6991	FKBP5
hsa_circ_0009082	0.03450	0.04731	0.1398	1.4312	3.0270	1.0026	3.3619	5.2175	5.4733	ADAMTS16
hsa_circ_0067996	0.03452	0.04731	2.4963	5.5904	5.6757	6.5277	4.9045	4.4215	4.6644	FNDC3B
hsa_circ_0042731	0.03487	0.04731	0.3423	1.7505	0.7735	2.0266	2.8053	3.3623	3.2406	PHF12
hsa_circ_0084887	0.03490	0.04731	0.4982	3.4516	3.0045	2.6463	4.0895	4.4026	3.6396	TMEM67
hsa_circ_0016946	0.03549	0.04741	0.4100	2.0742	2.2558	1.2093	2.7484	3.6061	3.1146	PCNXL2
hsa_circ_0056125	0.03594	0.04741	2.4529	4.2224	4.2365	4.0780	2.9771	3.2276	2.3043	MERTK
hsa_circ_0077995	0.03615	0.04741	4.7639	6.8270	7.8987	8.4742	4.8128	4.8189	6.5274	TNFAIP3
hsa_circ_0035018	0.03638	0.04741	2.6659	6.8960	7.3867	6.9612	5.1446	5.3399	6.2912	CKMT1B
hsa_circ_0055865	0.03647	0.04741	0.3476	3.8277	2.5467	3.2316	4.9088	4.9772	4.5329	NPAS2
hsa_circ_0008850	0.03681	0.04741	2.3518	6.4117	7.2368	7.2229	5.3880	5.4279	6.3025	---
hsa_circ_0075951	0.03708	0.04741	2.8812	7.4433	7.0311	8.2204	6.6974	5.4854	5.9244	HLA-DQA1
hsa_circ_0072107	0.03733	0.04741	4.5934	6.0713	4.3545	5.8546	3.0607	2.7164	4.0681	NPR3
hsa_circ_0032352	0.03739	0.04741	2.0339	6.8127	7.6105	7.3802	6.0765	5.9381	6.7068	KIAA0247
hsa_circ_0091701	0.03755	0.04741	2.4025	3.4102	3.8067	3.0836	1.8586	2.7805	1.7139	VMA21
hsa_circ_0032139	0.03764	0.04741	2.0471	4.4754	4.6045	5.2688	3.7533	4.0012	3.5953	HIF1A
hsa_circ_0042076	0.03775	0.04741	0.2902	2.5622	1.1134	1.8451	3.5276	3.7601	3.9167	GLP2R
hsa_circ_0079949	0.03777	0.04741	0.4453	6.7497	6.7868	6.0435	7.8149	8.0880	7.1339	GLI3
hsa_circ_0071767	0.03790	0.04741	0.3220	2.2244	0.7752	1.2588	2.8013	3.1828	3.4870	SRD5A1
hsa_circ_0051717	0.03810	0.04741	0.4316	2.8707	2.1163	1.8221	3.3656	3.8312	3.4125	LIG1
hsa_circ_0026401	0.03823	0.04741	4.2037	5.3375	3.8808	3.7903	2.2544	1.8831	2.9917	KRT6B
hsa_circ_0076153	0.03850	0.04741	5.9689	7.2449	8.9047	7.4325	6.1924	3.9459	5.5268	FKBP5
hsa_circ_0055863	0.03856	0.04741	0.4653	3.7872	3.7126	3.8380	5.0666	5.0859	4.3999	NPAS2
hsa_circ_0057350	0.03866	0.04741	0.4751	6.9195	6.8306	6.8191	7.4725	8.1919	8.0331	COL3A1
hsa_circ_0000353	0.03895	0.04752	3.2491	6.0080	6.5382	7.4178	4.7945	4.8328	5.4851	BIRC3
hsa_circ_0074580	0.03953	0.04799	2.3034	3.9751	3.6215	4.3085	2.5313	2.2710	3.3456	TNIP1

hsa_circ_0079950	0.03980	0.04808	0.4570	4.8424	4.6147	3.8626	5.8273	5.6748	5.3346	GLI3
hsa_circ_0032578	0.04011	0.04815	2.5119	5.1257	5.2480	4.8220	4.1746	3.0366	3.8099	LTBP2
hsa_circ_0042104	0.04059	0.04815	0.3448	1.5411	2.9882	2.1336	4.3140	3.6543	3.5477	MYOCD
hsa_circ_0056122	0.04072	0.04815	2.5571	5.4568	6.3027	5.4353	4.3751	4.3805	4.5477	MERTK
hsa_circ_0069155	0.04084	0.04815	2.1615	6.6979	5.9866	6.5392	5.0698	4.8673	5.8483	SH3TC1
hsa_circ_0049394	0.04093	0.04815	2.9865	5.8760	7.2094	6.8851	4.7981	4.8644	5.6962	LDLR
hsa_circ_0053875	0.04164	0.04815	0.4868	2.0502	2.9268	2.6598	3.4861	3.5945	3.7896	TTC27
hsa_circ_0019978	0.04174	0.04815	2.5573	5.1884	4.3436	5.5087	3.5744	3.3617	4.1546	ITPRIP
hsa_circ_0069160	0.04190	0.04815	4.0377	4.3161	4.7634	4.8849	2.5240	1.5568	3.3624	SH3TC1
hsa_circ_0026565	0.04196	0.04815	0.4781	4.0022	3.8014	3.7469	4.9184	5.2808	4.4352	ZNF740
hsa_circ_0061118	0.04200	0.04815	6.4647	2.6912	4.7695	4.6783	1.9657	0.5537	1.9279	SLCO4A1
hsa_circ_0000395	0.04259	0.04815	2.0995	4.3943	4.5474	4.1607	3.6768	3.3591	2.7276	SLC38A1
hsa_circ_0060372	0.04261	0.04815	0.4851	2.8454	2.0010	1.9960	3.3305	3.1521	3.6253	DHX35
hsa_circ_0044509	0.04268	0.04815	0.3443	9.3905	10.5676	9.1896	11.0614	11.9039	11.0248	COL1A1
hsa_circ_0035229	0.04277	0.04815	0.4971	1.9213	2.6224	1.7013	2.7796	3.2005	3.3905	TRPM7
hsa_circ_0046448	0.04282	0.04815	2.2020	3.1901	3.3491	3.3331	2.4541	1.5844	2.2840	TBCD
hsa_circ_0049386	0.04316	0.04830	3.3568	6.5562	8.0234	7.5921	5.1514	5.3123	6.4553	LDLR
hsa_circ_0044507	0.04358	0.04842	0.3434	9.3231	10.4877	9.0681	11.0121	11.7936	10.9617	COL1A1
hsa_circ_0068409	0.04375	0.04842	2.5552	4.4063	5.2548	4.2343	3.7006	3.5281	2.6016	IGF2BP2
hsa_circ_0005489	0.04411	0.04842	0.4158	1.9122	1.2225	2.3901	3.3861	2.9149	3.2131	FLCN
hsa_circ_0008474	0.04420	0.04842	0.2196	2.7093	1.7593	1.6523	3.0060	4.6042	4.7634	ADAMTS16
hsa_circ_0004654	0.04426	0.04842	2.4409	3.5165	4.1936	3.2232	3.0086	2.0832	1.8974	MOCOS
hsa_circ_0080211	0.04470	0.04848	2.3385	3.4520	4.4187	4.0384	2.8990	2.8330	2.6479	GRB10
hsa_circ_0019478	0.04498	0.04848	2.0260	3.2925	3.7592	3.6413	3.0369	2.0780	2.3919	ABCC2
hsa_circ_0044503	0.04524	0.04848	0.3634	9.4096	10.5349	9.1771	11.0265	11.7384	10.9971	COL1A1
hsa_circ_0008634	0.04533	0.04848	2.1530	7.2326	6.9830	6.7895	5.8287	5.3308	6.3733	IRAK3
hsa_circ_0049379	0.04543	0.04848	2.2460	7.2997	8.3370	7.8474	6.4146	6.3917	7.2034	LDLR
hsa_circ_0052833	0.04576	0.04848	0.3671	3.9128	4.9619	4.4568	6.2418	6.2424	5.1036	SDC1
hsa_circ_0047677	0.04590	0.04848	2.0746	5.0771	5.0981	4.8600	4.4004	3.8623	3.4748	MYO5B
hsa_circ_0026362	0.04618	0.04848	3.7783	6.4217	5.6048	4.8405	3.5599	3.7983	4.1249	KRT7
hsa_circ_0061123	0.04623	0.04848	4.4013	5.1181	6.8603	6.6488	4.5838	3.6004	4.3908	SLCO4A1
hsa_circ_0031261	0.04630	0.04848	0.2738	2.5124	0.7572	1.6982	3.9957	3.6010	3.4308	AJUBA
hsa_circ_0089830	0.04703	0.04880	2.0505	5.0365	4.3035	5.2056	3.5649	3.8357	4.1311	PRKX

hsa_circ_0054693	0.04729	0.04880	2.2285	6.0561	5.9443	6.7618	4.5950	4.9443	5.6789	REL
hsa_circ_0077996	0.04749	0.04880	3.7992	7.0040	8.0533	8.7614	5.9626	5.5373	6.7698	TNFAIP3
hsa_circ_0004761	0.04757	0.04880	0.4806	3.7100	2.9082	2.8722	4.2468	4.1569	4.4104	DENND2A
hsa_circ_0026805	0.04760	0.04880	0.2135	7.7153	7.2360	7.6762	9.4922	10.5435	8.7684	RPS26
hsa_circ_0000143	0.04813	0.04912	2.2362	3.5704	3.7418	4.5515	3.0703	2.2842	3.0893	CD1D
hsa_circ_0069161	0.04835	0.04912	3.1993	3.6074	4.0448	4.6830	1.6579	1.9976	3.2999	SH3TC1
hsa_circ_0078342	0.04851	0.04912	5.4654	3.1869	5.1949	4.9724	2.1871	2.7296	1.5488	IPCEF1
hsa_circ_0049665	0.04947	0.04973	2.1058	3.9157	4.2580	4.2779	3.5763	2.9459	2.5435	TRMT1
hsa_circ_0044512	0.04971	0.04973	0.3519	9.3780	10.5618	9.0905	11.0381	11.8410	10.9397	COL1A1
hsa_circ_0049376	0.04973	0.04973	2.7195	6.3929	7.5975	7.3433	5.5457	5.6506	6.0198	LDLR

Table S3 Primers and probes used in the study

Gene	Sequence	Product length （bp）
hsa_circ_0004846	5'-CCAGCACAAGTACAAGAATCATTA-3'<br>3'-AGAGTGAGCCAGGATTTTGGG-5'	155
hsa_circ_0031995	5'-ACCAGTGGATTCGTCAGCTC -3'<br>3'-AGCTTCTGCACCTGCTGTTT -5'	131
hsa_circ_0035019	5'-TATGCTGCAGATAGCCGCTT -3'<br>3'-TATCAAAGACACCGCCTGTAGC -5'	103
hsa_circ_0002612	5'- CTTCAGGAAGTGGTGGGAGT -3'<br>3'- TTCAGCAAAGGCAGATGAGTT -5'	160
hsa_circ_0002012	5'- AATAGCATGCCAAGCCGCAC -3'<br>3'- AGGGAGTTGGATTGCCCAAA -5'	124
hsa_circ_0024182	5'- AGCTTGCTTGTCTGTATGCC -3'<br>3' -AGGAGCCTGTCTCTTGCTTG -5'	118
hsa_circ_0031990	5'- GCAGTTCACACGCGTCAT -3'<br>3'- GACATCCATCCATGTCCTAGA -5'	126
hsa_circ_0035016	5'- TCTACTTGCTCTGGACCTGC -3'<br>3'- TGCAGTTGTTGTGCTTTCGG -5'	107
hsa_circ_0031996	5'- GTGCACACTTCCCCACAGAA -3'<br>3'- GACTGAGCTGTGGGTGCG -5'	106
hsa_circ_0027396	5'- GTGAAGGCATAATGGGCTGT -3'<br>3'- TAGATGGTGCGGATGGAATC -5'	122
hsa_circ_0023599	5'- GAAGTCACTGAAGGCCAGGA -3'<br>3'- TTGGCATTAATGGTGCACAG -5'	132
hsa_circ_0023595	5'- GAGCGACTGGGAATCCACTT -3'<br>3'- TGACTTCACAGGAAGGATCTCG -5'	179
hsa_circ_0055859	5'- CTGGGAACCTCAGGCTATGAC -3'<br>3'- CCGACGCTTCTTCTCAGACT -5'	104
hsa_circ_0087875	5'- CCTCGCCTATGATTGGCACT -3'<br>3'- CTGAACTCCAGGGTCCGAAAT -5'	179
hsa_circ_0083215	5'- TGAAGACCCAGACAAGGATGA -3'<br>3'- CGCACGGCTGGTTCTATAGTT -5'	159
CircANRIL	5'- TGACAGTGTCCCTTTTGATGAGA -3'<br>3'- GGAAAGGATTCCAGCACACCT -5'	195
hWnt1	5'- CCGATGGTGGGGTATTGTGA -3'<br>3'- TCCCCGGATTTTGGCGTATC -5'	137
hSAMD4A	5' - AGAAATCGAGGCTTTGGGCA -3'<br>3' - CCGAGAGGGGATCTGGTACT -5'	102
hCCND3	5'- GAGGTGCAATCCTCTCCTCG -3'<br>3'- GCTGCTCCTCACATACCTCC -5'	145
hEZH2	5'- ACATCCTTTTCATGCAACACC -3'<br>3'- GCTCCCTCCAAATGCTGGTA -5'	105
hSIRT1	5'- GGGCTGCGGTTCCTACTG -3'<br>3'- AAGGTTATCTGGCTGCTGCG -5'	104
hSOX4	5'- GCACTAGGACGTCTGCCTTT -3'<br>3'- ACACGGCATATTGCACAGGA -5'	92
hEID1	5'- CCGAAGAACTCGGCTGTGAT -3'<br>3'- AGTTGGGTCCCTCCTCAAGT -5'	89
hWnt10b	5'- CTGACAAGGGGACAGAACCC -3'<br>3'- TCAGGACCTCCAGTGGTTTG -5'	117
hPPAR- γ	5'- CGAGAGTCAGCCTTTAACGAAATG -3'<br>3'- AGGAGTGGGAGTGGTCTTCC -5'	115
hCEBP- α	5'- TATAGGCTGGGCTTCCCCTT -3'<br>3'- AGCTTTCTGGTGTGACTCGG -5'	94

hGAPDH	5’- GAAAGCCTGCCGGTGACTAA -3’<br>3’- GCCCAATACGACCAAATCAGAG -5’	150
mmu_circ_0000529	5’- GCAAGCACGAGAATCATTAACCA -3’<br>3’- TTCGATAGAGTGTGCCAGGAT -5’	156
mSAMD4	5’ - GAGCTGCGTGGAGTAGTGAT -3’<br>3’ - AAATCCCTCGCAAGGCACAA -5’	164
mEZH2	5’- TCCATGCAACACCCAACACA -3’<br>3’- CCTTAGCTCCCTCCAGATGC -5’	102
mPPAR- γ	5’- CAGGTCAGAGTCGCCCCG -3’<br>3’- CCGGTCACCTTGTCGTCACA -5’	127
mC/EBP- α	5’- AATGGCAGTGTGCACGTCTA -3’<br>3’- CCCCAGCCGTTAGTGAAGAG -5’	109
mGAPDH	5’- CCCTTAAGAGGGATGCTGCC -3’<br>3’- TACGGCCAAATCCGTTCACA -5’	124

circRIP probes

circSAMD4A	5’- TGTTGCCATTGGTTAATGATTCTTGTACTTGTGCTGGTTCCAAT -3’-biotin
Control	5’-CTAAGACTAGGTGTTCGGAGGGAAAACAAAAAGAGATATCAGAA -3’-biotin

miRNA probes

miR-138-5p	biotin-5’- CGGCCTGATTCACAACACCAGCT -3’-biotin

FISH probes

circSAMD4A	digoxin-5’- CCTGTTGCCATTGGTTAATGATTCTTGTACTTGTGCTGGTTCC -3’-digoxin
Negative Control	5’-TAAGACTAGGTGTTCGGAGGGAAAACAAAAAGAGATATCAGAA -3’-biotin
GAPDH	digoxin-5’- AGGCGCCCAATACGACCAAATCAGAGAATAATCTAGGAAAAGCA -3’-digoxin

siRNAs

circSAMD4A	5’- AGCACAAGUACAAGAAUCAUU -3’
EZH2	5’- ACAUACUCUUUACUUCAUCAG -3’
EID-1	5’- UCUUGUUCUCAGAAACAUCUU -3’

ISH probes

circSAMD4A	digoxin-5’- TGGTTAATGATTCTTGTACTTGTG -3’-digoxin

Table S4 CircSAMD4A expression in obese sample

Clinical or molecular feature	CircSMAD4A(30) over-expression	CircSMAD4A(30) down-expression	*P*
Age (y)	25±3.7	31±5.6	*0.001*
Gender (M/F)	7/23	7/23	
BMI (kg/m^2)	40.8±6.56	35.5±4.32	*<0.001*
Systolic BP (mm Hg)	127.3±11.2	132±17.3	*0.202*
Diastolic BP (mm Hg)	77.9±7.77	84.8±12.3	*0.013*
Diabetes	11%(3/30)	19%(6/30)	*0.286*
Hyperuricemia (HUA)	29%(8/30)	13%(4/30)	*0.203*
Hyperlipemia (HLP)	54%(17/30)	63%(18/30)	*0.798*
Obstructive sleep apnea syndrome (OSAS)	75%(22/30)	56%(17/30)	*0.182*
Hyperinsulinemia (HINS)	4%(1/30)	19%(6/30)	*0.045*

Table S5 Standardized ORs evaluating the risk of patients in non-remission

Characteristics	Risk of non-remission vs remission		
	OR	95% CI	*P*
Age (mean±SD)	1.029	0.874-1.213	*0.730*
Gender	2.667	0.415-17.120	*0.301*
Systolic BP	1.043	0.971-1.120	*0.250*
Diastolic BP	1.016	0.938-1.100	*0.705*
Diabetes	0.673	0.107-4.243	*0.673*
HUA	2.307	0.410-12.996	*0.343*
HLP	0.188	0.039-0.912	*0.038*
OSAS	0.567	0.114-2.809	*0.487*
HINS	2.064	0.173-24.596	*0.567*
CircSAMD4A	13.795	2.249-84.617	*0.005*

Table S6 miRNAs that might potentially bind to circSAMD4A

miRNAs	Score
hsa-miR-133a-5p	-0.164
hsa-miR-138-5p	-0.160
hsa-miR-942-3p	-0.160
hsa-miR-6131	-0.146
hsa-miR-7152-5p	-0.126
hsa-miR-6874-3p	-0.116
hsa-miR-1245b-3p	-0.111
hsa-miR-148b-5p	-0.098
hsa-miR-4717-3p	-0.098
hsa-miR-4422	-0.042

Supplementary methods

Definition of remission and non-remission in obese patients

Percentage excess weight loss (%EWL)= 100% × (baseline weight−last weight) / (baseline weight－ideal weight) [1], Baseline weight refers to preoperative weight, last weight equal to the current weight. In China, ideal weight (kg) for men as ideal weight = height (cm)-105, and for women as ideal weight = height (cm)-100 [2].

Reinhold et al [3] defined EWL% ≥75% as very good effect. As the above method is simple and practicable, it has been quoted widely and has become a standard. Therefore we defined EWL% ≥75% as remission, and EWL% ≤75% as non-remission.

References

1. Weigley ES. Average? Ideal? Desirable? A brief overview of height-weight tables in the United States. J Am Diet Assoc. 1984; 84: 417-23.
2. Nowaczyk P, Murawa D, Połom K, Waszyk-Nowaczyk M, Spychała A, Michalak M, et al. Analysis of sentinel lymph node biopsy results in colon cancer in regard of the anthropometric features of the population and body composition assessment formulas. Langenbecks Arch Surg. 2012; 397: 779-86.
3. Reinhold RB. Critical analysis of long term weight loss following gastric bypass. Surg Gynecol Obstet. 1982; 155: 385-94.
